# Diversity of HLA Class I and Class II blocks and conserved extended haplotypes in Lacandon Mayans

**DOI:** 10.1038/s41598-020-58897-5

**Published:** 2020-02-24

**Authors:** Rodrigo Barquera, Joaquin Zuniga, José Flores-Rivera, Teresa Corona, Bridget S. Penman, Diana Iraíz Hernández-Zaragoza, Manuel Soler, Letisia Jonapá-Gómez, Kalyan C. Mallempati, Petra Yescas, Adriana Ochoa-Morales, Konstantinos Barsakis, José Artemio Aguilar-Vázquez, Maricela García-Lechuga, Michael Mindrinos, María Yunis, Luis Jiménez-Alvarez, Lourdes Mena-Hernández, Esteban Ortega, Alfredo Cruz-Lagunas, Víctor Hugo Tovar-Méndez, Julio Granados, Marcelo Fernández-Viña, Edmond Yunis

**Affiliations:** 1 Department of Archaeogenetics, Max Planck Institute for the Science of Human History (MPI-SHH), Jena, Germany; 2 Laboratory of Molecular Genetics, Escuela Nacional de Antropología e Historia (ENAH), Mexico City, Mexico; 3 Department of Immunology, Instituto Nacional de Enfermedades Respiratorias Ismael Cosío Villegas (INER), Mexico City, Mexico; 4 Tecnologico de Monterrey, Escuela de Medicina y Ciencias de la Salud, Mexico City, Mexico; 5 Clinical Laboratory of Neurodegenerative Diseases, Instituto Nacional de Neurología y Neurocirugía “Manuel Velasco Suárez”, Mexico City, Mexico; 6 University of Warwick, School of Life Sciences, Coventry, United Kingdom; 7 Immunogenetics Unit, Técnicas Genéticas Aplicadas a la Clínica (TGAC), Mexico City, Mexico; 8 Department of Transplantation, Instituto Nacional de Ciencias Médicas y Nutrición Salvador Zubirán (INCMSZ), Mexico City, Mexico; 9 Public Health State Laboratory for Chiapas, Tuxtla Gutierrez, Chiapas, Mexico; 10 Histocompatibility, Immunogenetics and Disease Profiling Laboratory, Stanford Blood Center, Palo Alto, CA USA; 11 Biology Department, University of Crete, Heraklion, Greece; 12 Department of Neurogenetics and Molecular Biology, Instituto Nacional de Neurología y Neurocirugía “Manuel Velasco Suárez”, Mexico City, Mexico; 13 Department of Pathology, Stanford University, CA USA; 14 Clinical Analysis Laboratory, Unidad Médica Familiar (UMF) No. 23, Instituto Mexicano del Seguro Social (IMSS), Tuxtla Gutiérrez, Chiapas Mexico; 15 Stanford Genome Technology Center, Palo Alto, CA USA; 16 Department of Cancer Immunology and Virology, Dana Farber Cancer Institute, Harvard Medical School, Boston, MA USA; 17 The William Harvey Research Institute, Barts and London School of Medicine, Queen Mary University of London, London, United Kingdom

**Keywords:** Immunogenetics, Immunogenetics

## Abstract

Here we studied HLA blocks and haplotypes in a group of 218 Lacandon Maya Native American using a high-resolution next generation sequencing (NGS) method. We assessed the genetic diversity of HLA class I and class II in this population, and determined the most probable ancestry of Lacandon Maya HLA class I and class II haplotypes. Importantly, this Native American group showed a high degree of both HLA homozygosity and linkage disequilibrium across the HLA region and also lower class II HLA allelic diversity than most previously reported populations (including other Native American groups). Distinctive alleles present in the Lacandon population include HLA-A*24:14 and HLA-B*40:08. Furthermore, in Lacandons we observed a high frequency of haplotypes containing the allele HLA-DRB1*04:11, a relatively frequent allele in comparison with other neighboring indigenous groups. The specific demographic history of the Lacandon population including inbreeding, as well as pathogen selection, may have elevated the frequencies of a small number of HLA class II alleles and DNA blocks. To assess the possible role of different selective pressures in determining Native American HLA diversity, we evaluated the relationship between genetic diversity at *HLA-A*, *HLA-B* and *HLA-DRB1* and pathogen richness for a global dataset and for Native American populations alone. In keeping with previous studies of such relationships we included distance from Africa as a covariate. After correction for multiple comparisons we did not find any significant relationship between pathogen diversity and HLA genetic diversity (as measured by polymorphism information content) in either our global dataset or the Native American subset of the dataset. We found the expected negative relationship between genetic diversity and distance from Africa in the global dataset, but no relationship between HLA genetic diversity and distance from Africa when Native American populations were considered alone.

## Introduction

The human major histocompatibility complex (MHC) is located within chromosomal region 6p21.3 and spans at least 3.4 Mb of DNA containing more than 400 genes. Human leukocyte antigen (HLA) loci are mapped within the MHC region as well as other immune related genes and pseudogenes^1^. The genetic diversity of MHC genes results from selective pressures including functional adaptation to pathogens^2–8^ with some peptide-HLA complexes being more effective in eliciting an immune response than others^4^. It seems likely that individuals with greater diversity at their HLA loci would have a greater chance of survival in pathogen-enriched environments^5^, but there is debate as to whether simple heterozygote advantage or more complex host-pathogen co-evolutionary processes are responsible for the diversity of human HLA alleles generally^9–15^. In addition to their direct role in immune responses against pathogens, HLA molecules are crucial genetic markers to study the genetic diversity of populations in the context of disease susceptibility and allotransplantation^3–5,16–20^.

One of the most important characteristics of the MHC region is a high degree of non-random associations between inherited alleles, known as linkage disequilibrium (LD)^21,22^. Extensive studies in different populations have described the existence of extended haplotypes or blocks and other relatively fixed genetic fragments within the human MHC^17,23^. Specific DNA blocks containing two or more MHC loci are often haplospecific for particular conserved extended haplotypes (CEHs)^17^. The frequency of CEHs and specific block combinations varies between major ancestral groups and/or in different continental populations, and these variations in the frequency of CEHs and blocks can be used as measurements of genetic diversity of the MHC^17,18,23^. It has further been suggested that a specific form of linkage disequilibrium - non-overlapping combinations between physically separate HLA loci - could be a signal of pathogen selection within HLA system^24^. Assessing the presence of such non-overlapping associations thus arises as a possibility to assess pathogen driven selection imprinted in the genetic structure of HLA system within populations.

Several studies have described HLA class I and class II alleles and haplotypes in different Native American groups from Mexico^25–32^ and Latin America^33–36^, including Lacandons^37^. The study of HLA diversity in Native Americans is relevant because evidence suggests that balancing selection at different HLA loci may be involved in the prevalence of inflammatory or infectious diseases in these populations^38–43^. Also, numerous examples of novel alleles have been reported in Native Americans^44–46^, suggesting that pathogen-driven selection of new mutations could be critical in the adaptation to endemic pathogens, particularly after migration^4,5^.

Previous studies of the Lacandon population of Chiapas State have described the genetic variability of low resolution HLA class II^37^, blood groups^47^ and single nucleotide polymorphisms (SNPs) of Cytochrome P450 2D6^48^. Individuals from the Lacandon population were also included in a study of genomic variation across Mexico^49^. However, no studies have been published about the immunogenetic diversity and the possible ancestral origin of HLA conserved extended haplotypes (CEH) in a representative group of Lacandon Maya.

**Figure 1:**
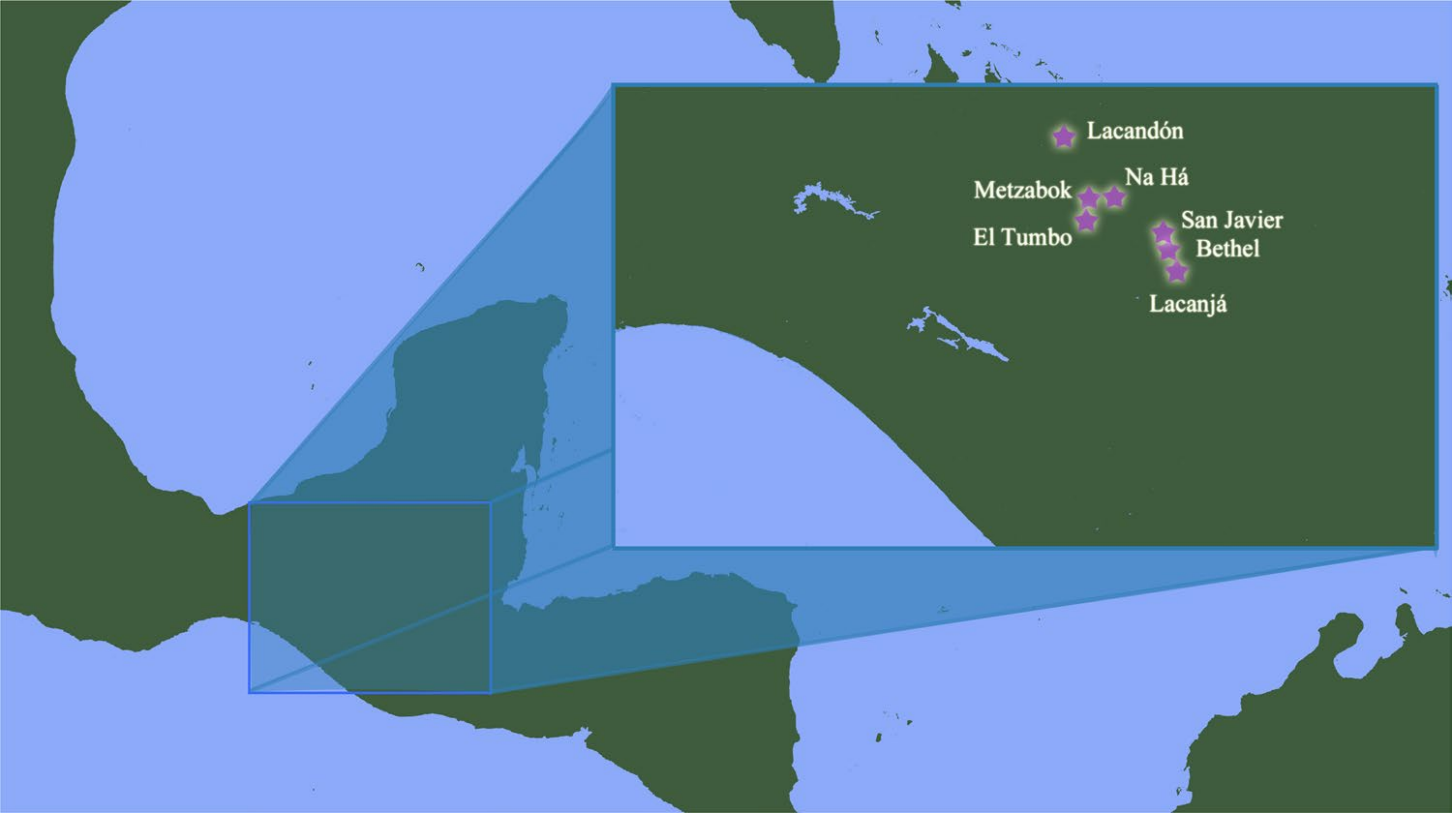
Locations where the samples were obtained in Chiapas, Mexico. The communities where the samples were taken are represented by a star.

The Lacandon or *Hach Winik* (“the real people”) are descendants of ancient Mayan civilizations, which have been considered to have reached their cultural apex in Mesoamerica c. AD 800^50,51^. Their settlements are mainly distributed in the Lacandon Jungle (Fig. 1), which stretches from the State of Chiapas, Mexico, into Guatemala and into the southern part of the Yucatán Peninsula^52,53^. The historical landscape of Lacandon Maya includes multiple genocides related with Spaniard conquest, land disputes as well as novel infections such as smallpox, yellow fever and influenza, and a high degree of inbreeding that has resulted in high prevalence of certain genetic conditions, including albinism^51,54,55^.

The aim of this study is to analyze the immunogenetic diversity and the ancestral origin of HLA CEH in Lacandon Maya using next generation high-resolution HLA typing methods, and to use such information to assess if there are signatures of evolutionary forces modifying the expected diversity within the HLA system. We also assess whether a correlation exists between the genetic diversity (as measured by the polymorphism informative content, PIC) at class I (*HLA-A*, *-B*) and class II (*-DRB1*) loci in Native Americans (including Lacandon Mayans) and a) pathogen and viral richness, and b) the geographic distance from Africa.

## Results

### HLA allele and haplotype frequencies within the Lacandon population

HLA allele frequencies were obtained by analyzing 458 haplotypes from 229 non-first degree related Lacandon Maya individuals genotyped by next generation sequencing (NGS) technologies. Complete haplotypes could be obtained for 218 individuals for a total of 436 haplotypes. The most frequent class I haplotypes, all with haplotypic frequencies (H.F.) higher than 5% were HLA-A*31:01~B*40:02~C*03:04 [Native American Most probable ancestry (MPA)], A*02:06~B*35:01~C*07:02 (Native American MPA), A*68:03~B*35:01~C*07:02 (Not previously reported), A*24:02~B*35:12~C*04:01 (Native American MPA), A*68:01~B*40:08~C*03:04 (previous reports only include mixed-ancestry populations), A*68:03~B*39:05~C*07:02 (previous reports only include mixed-ancestry populations), and A*68:03~B*39:05~C*07:02 (previous reports only include mixed-ancestry populations). Only two *HLA-A*~*B*~*C*~*DRB1*~*DRB3*/4/5~*DQB1*~*DQA1*~*DPA1*~*DPB1* haplotypes exhibited frequencies greater that 5%: A*31:01~B*40:02~C*03:04-DRB1*04:11~DRB4*01:03~DQB1*03:02~DQA1*03:01~DPA1*01:03~DPB1*04:02 (H.F. = 0.0633) and A*31:01~B*40:02~C*03:04~DRB1*04:11~DRB4*01:01~DQB1*03:02~DQA1*03:01~DPA1*01:03~DPB1*04:02 (H.F. = 0.0502). These two high frequency haplotypes differ only in their *HLA-DRB*4 allele. The complete frequencies of *HLA-A*, *-B*, *-C*, *-DRB1*, *-DRB3*/*4*/5, *-DQA1*, *-DQB1*, *-DPA1*, and *-DPB1* alleles in Lacandon Maya individuals are detailed in Table 1 (class I) and Table 2 (class II). *HLA-B*~*C* blocks present in Lacandon Maya are listed in Table 3, while the extension of the *HLA-B*~*C* block to the *HLA-A* gene is provided in Supplementary Table 2. HLA class II haplotypic diversity (*HLA-DRB1*~*DRB3*/*4*/*5*~*DQA1*~*DQB1*~*DPA1*~*DPB1*) is listed in Table 4. The CEHs in Lacandons are listed in Table 5 and their extension to *HLA-A* is shown in Table 6. Table 7 shows the complete class I/class II haplotypes for our Lacandon Mayans sample. Aggregate block frequency (ABF) for each ancestry can be found in Tables 3 to 6. The calculation of ABF is a previously reported and validated approach to estimate the diversity and contribution of precisely described HLA blocks of specific ancestries^17,56^.

**Table 1:** Allelic frequencies of HLA class I (*HLA-A*, *-B* and *-C*) in 218 Lacandon Native Americans.

HLA-A protein	*HLA-A* allele	A.F.	HLA-B protein	*HLA-B* allele	A.F.	HLA-C protein	*HLA-C* allele	A.F.
A*24:02		**0.1904**	B*40:02		**0.2638**	C*07:02		**0.3784**
	A*24:02:01:01	0.1790		B*40:02:01	0.2764		C*07:02:01:01	0.3732
	A*24:02:01:02 L	0.0023	B*35:01		**0.2156**		C*07:02:01:03	0.0052
A*31:01		**0.1697**		B*35:01:01:02	0.2133	C*03:04		**0.2798**
	A*31:01:02:01	0.1651	B*39:05		**0.1651**		C*03:04:01:01	0.0677
	A*31:01:13	0.0046		B*39:05:01	0.1562		C*03:04:01:02	0.2083
A*68:01		**0.1445**	B*35:12		**0.1101**	C*04:01***		**0.1789**
	A*68:01:02	0.0046		B*35:12:01	0.1101		C*04:01:01:01	0.1789
	A*68:01:02:01	0.1359	B*40:08		**0.0665**	C*15:02***		**0.0436**
A*02:06		**0.1399**		B*40:08	0.0459		C*15:02:01:01	0.0046
	A*02:06:01:01	0.1157	B*18:01		**0.0275**		C*15:02:01:02	0.0390
A*68:03		**0.1376**		B*18:01:01:01	0.0023	C*07:01		**0.0344**
	A*68:03:01	0.1070		B*18:01:01:02	0.0252		C*07:01:01:01	0.0321
A*02:01		**0.0940**	B*52:01***		**0.0161**		C*07:01:02	0.0023
	A*02:01:01:01	0.0764		B*52:01:02	0.0153	C*03:05		**0.0183**
A*24:14		**0.0550**	B*39:01		**0.0138**	C*01:02		**0.0161**
A*68:05		**0.0275**	B*39:06		**0.0138**		C*01:02:01	0.0046
A*29:02		**0.0115**		B*39:06:02	0.0138	C*03:03		**0.0115**
	A*29:02:01:01	0.0115	B*39:08		**0.0138**		C*03:03:01	0.0104
A*03:01		**0.0069**		B*39:08	0.0138	C*16:01		**0.0115**
	A*03:01:01:01	0.0069	B*44:03***		**0.0115**		C*16:01:01:01	0.0069
A*33:01		**0.0046**		B*44:03:01:01	0.0115	C*08:02		**0.0092**
	A*33:01:01	0.0023	B*35:43		**0.0115**		C*08:02:01:01	0.0023
A*30:01		**0.0046**		B*35:43:01	0.0069	C*15:04***		**0.0046**
	A*30:01:01	0.0046	B*14:02		**0.0092**		C*15:04:01	0.0046
A*68:02		**0.0046**		B*14:02:01:01	0.0023	C*05:01***		**0.0023**
	A*68:02:01:01	0.0046		B*14:02:02	0.0046		C*05:01:01:02	0.0023
A*01:01		**0.0023**	B*51:01***		**0.0092**	C*06:02***		**0.0023**
A*26:01		**0.0023**		B*51:01:01:01	0.0092		C*06:02:01:01	0.0023
	A*26:01:01:01	0.0023	B*07:02		**0.0069**	C*08:01		**0.0023**
A*30:02		**0.0023**		B*07:02:01	0.0069		C*08:01:01	0.0023
	A*30:02:01:01	0.0023	B*35:17		**0.0069**	C*12:02		**0.0023**
A*33:03	A*33:03:01	**0.0023**		B*35:17:01	0.0069	C*15:15***		**0.0023**
			B*49:01***		**0.0069**	C*17:01***		**0.0023**
				B*49:01:01	0.0069		C*17:01:01:02	0.0023
			B*35:08		**0.0046**			
				B*35:08:01	0.0046			
			B*39:02		**0.0046**			
				B*39:02:02	0.0046			
			B*40:11		**0.0046**			
				B*40:11:01	0.0046			
			B*53:01***		**0.0046**			
				B*53:01:01	0.0046			
			B*13:02***		**0.0023**			
				B*13:02:01	0.0023			
			B*35:14		**0.0023**			
				B*35:14:01	0.0023			
			B*39:39		**0.0023**			
				B*39:39:01	0.0023			
			B*15:01		**0.0023**			
			B*44:02***		**0.0023**			
				B*44:02:01:01	0.0023			
			B*42:02		**0.0023**			
				B*42:02:01:02	0.0023			

A.F.: Allele frequency. HLA-B alleles marked with *** are classified as HLA-Bw4 alleles^132^. HLA-C alleles marked with *** are classified as HLA-C2 alleles^91^.

**Table 2:** Allelic frequencies of HLA class II genes (*HLA-DRB1*, -*DRB3*/*4*/*5*, -*DQA1*, -*DQB1*, -*DPA1* and -*DPB1*) in 218 Lacandon Native Americans.

HLA-DRB1 protein	*HLA-DRB1* allele	A.F.	HLA-DRB3/4/5 protein	*HLA-DRB3*/*4*/*5* allele	A.F.	HLA-DQA1 protein	*HLA-DQA1* allele	A.F.	HLA-DPA1 protein	*HLA-DPA1* allele	A.F.
DRB1*04:11		**0.3624**	DRB4*01:01		**0.3493**	DQA1*03:01		**0.6950**	DPA1*01:03		**0.9014**
	DRB1*04:11:01	0.3555	DRB4*01:03		**0.3472**		DQA1*03:01:01	0.6950		DPA1*01:03:01:01	0.0373
	DRB1*04:11:04	0.0046		DRB4*01:03:01:01	0.3403	DQA1*05:05		**0.1055**		DPA1*01:03:01:02/03	0.1196
	DRB1*04:11:18	0.0023		DRB4*01:03:02e1	0.0069		DQA1*05:05:01:01	0.1055		DPA1*01:03:01:04	0.7376
DRB1*04:07		**0.2385**	DRB3*01:01		**0.1027**	DQA1*05:03		**0.0872**		DPA1*01:03:03	0.0046
	DRB1*04:07:01	0.2293		DRB3*01:01:02:01	0.1004	DQA1*04:01		**0.0711**		DPA1*01:03:04	0.0023
DRB1*04:04		**0.0711**		DRB3*01:01:02:02	0.0023		DQA1*04:01:01	0.0688	DPA1*02:02		**0.0665**
	DRB1*04:04:01	0.0688	DRB5*02:02		**0.0633**		DQA1*04:01:02:01	0.0023		DPA1*02:02:02	0.0487
DRB1*16:02		**0.0642**		DRB5*02:02e1	0.0633	DQA1*01:01		**0.0138**	DPA1*02:01		**0.0252**
	DRB1*16:02:01	0.0642	DRB3*02:02		**0.0373**		DQA1*01:01:02	0.0023		DPA1*02:01:01	0.0206
DRB1*14:02		**0.0573**		DRB3*02:02:01:01	0.0327	DQA1*02:01		**0.0069**	DPA1*01:11		**0.0023**
	DRB1*14:02:01	0.0590		DRB3*02:02:01:02	0.0046	DQA1*01:02		**0.0069**	DPA1*01:12		**0.0023**
DRB1*08:02		**0.0596**	DRB4*01:05		**0.0092**		DQA1*01:02:01:01	0.0023	DPA1*02:03		**0.0023**
	DRB1*08:02:01	0.0550	DRB5*01:01		**0.0046**		DQA1*01:02:01:03	0.0023	**HLA-DPB1 protein**	***HLA-DPB1 allele***	**A.F**.
	DRB1*08:02:03	0.0046	DRB3*03:01		**0.0023**		DQA1*01:02:01:04	0.0023			
DRB1*14:06		**0.0390**				DQA1*03:03		**0.0046**	DPB1*04:02		**0.7775**
	DRB1*14:06:01	0.0371					DQA1*03:03:01	0.0046		DPB1*04:02:01:01	0.0399
DRB1*11:04		**0.0275**				DQA1*05:01		**0.0046**		DPB1*04:02:01:02	0.7376
	DRB1*11:04:01	0.0275					DQA1*05:01:02	0.0046	DPB1*05:01		**0.0665**
DRB1*04:03		**0.0206**				DQA1*01:03		**0.0023**		DPB1*05:01:01	0.0577
	DRB1*04:03:01	0.0183					DQA1*01:03:01:02	0.0023	DPB1*04:01		**0.0619**
	DRB1*04:03:02	0.0023				DQA1*01:05		**0.0023**		DPB1*04:01:01:01	0.0619
DRB1*08:01		**0.0092**					DQA1*01:05:01	0.0023	DPB1*02:01		**0.0390**
DRB1*01:02		**0.0069**				**HLA-DQB1 protein**	***HLA-DQB1 allele***	**A.F**.		DPB1*02:01:02	0.0286
DRB1*13:03		**0.0069**							DPB1*03:01		**0.0183**
	DRB1*13:03:01	0.0069				DQB1*03:02		**0.6950**		DPB1*03:01:01	0.0137
DRB1*01:01		**0.0046**					DQB1*03:02:01	0.6756	DPB1*14:01		**0.0092**
DRB1*15:01		**0.0046**					DQB1*03:02:02	0.0194		DPB1*14:01:01	0.0092
DRB1*04:05		**0.0023**				DQB1*03:01		**0.1927**	DPB1*11:01		**0.0069**
DRB1*04:10		**0.0023**					DQB1*03:01:01:01	0.1593		DPB1*11:01:01	0.0069
	DRB1*04:10:01	0.0023					DQB1*03:01:01:03	0.0334	DPB1*17:01		**0.0069**
DRB1*04:17		**0.0023**				DQB1*04:02		**0.0711**		DPB1*17:01	0.0069
DRB1*04:51		**0.0023**					DQB1*04:02:01	0.0711	DPB1*10:01		**0.0046**
DRB1*04:91		**0.0023**				DQB1*05:01		**0.0161**		DPB1*10:01:01	0.0046
DRB1*04:154		**0.0023**					DQB1*05:01:01:01	0.0046	DPB1*105:01		**0.0046**
DRB1*07:01		**0.0023**					DQB1*05:01:01:02	0.0023	DPB1*126:01		**0.0046**
	DRB1*07:01:01:01	0.0023				DQB1*02:02		**0.0069**	DPB1*01:01		**0.0023**
DRB1*10:01		**0.0023**					DQB1*02:02:01:01	0.0069		DPB1*01:01:01	0.0023
DRB1*11:18		**0.0023**				DQB1*03:04		**0.0069**	DPB1*51:01		**0.0023**
DRB1*11:42		**0.0023**					DQB1*03:04:01	0.0046			
DRB1*12:01		**0.0023**					DQB1*03:04:02	0.0023			
	DRB1*12:01:01	0.0023				DQB1*06:03		**0.0046**			
DRB1*13:01		**0.0023**				DQB1*03:03		**0.0023**			
	DRB1*13:01:01	0.0023				DQB1*06:02		**0.0023**			
DRB1*13:02		**0.0023**				DQB1*06:04		**0.0023**			
DRB1*14:41		**0.0023**									

A.F.: Allele frequency.

**Table 3:** *HLA-B*~*C* blocks* in 218 Lacandon Native Americans.

Block	H.F.	n (N = 218)	Δ′	*p*	*t*
B*40:02~C*03:04	0.2024	88	0.6876	<0.0001	22.5
B*35:01~C*07:02	0.1656	72	0.6320	<0.0001	14.4
B*39:05~C*07:02	0.1610	70	0.9566	<0.0001	19.3
B*35:12~C*04:01	0.1058	46	0.9505	<0.0001	15.4
B*40:08~C*03:04	0.0667	29	1.0000	<0.0001	11.4
B*35:01~C*04:01	0.0483	21	0.0656	0.2187	1.9
B*40:02~C*15:02	0.0414	18	0.9279	<0.0001	8.5
B*18:01~C*07:01	0.0276	12	1.0000	<0.0001	7.4
B*40:02~C*03:05	0.0184	8	1.0000	<0.0001	5.7
B*39:01~C*07:02	0.0138	6	1.0000	0.0015	4.9
B*39:06~C*07:02	0.0138	6	1.0000	0.00153	4.9
B*39:08~C*07:02	0.0138	6	1.0000	0.00153	4.9
B*35:43~C*01:02	0.0115	5	1.0000	<0.0001	4.5
B*44:03~C*16:01	0.0115	5	1.0000	<0.0001	4.5
B*52:01~C*03:03	0.0115	5	1.0000	<0.0001	4.5
B*14:02~C*08:02	0.0092	4	1.0000	<0.0001	4.0
B*07:02~C*07:02	0.0069	3	1.0000	0.02555	3.4
B*35:17~C*04:01	0.0069	3	1.0000	0.0002	3.5
B*49:01~C*07:01	0.0069	3	1.0000	<0.0001	3.5
B*35:08~C*04:01	0.0046	2	1.0000	0.0022	2.8
B*35:12~C*07:02	0.0046	2	−0.8918	<0.0001	−7.4
B*39:02~C*03:04	0.0046	2	1.0000	0.02502	2.8
B*39:05~C*04:01	0.0046	2	−0.8467	0.00023	−7.2
B*40:11~C*03:04	0.0046	2	1.0000	0.02502	2.8
B*51:01~C*15:04	0.0046	2	1.0000	<0.0001	2.8
B*53:01~C*04:01	0.0046	2	1.0000	0.0022	2.8
B*13:02~C*06:02	0.0023	1	1.0000	<0.0001	2.0
B*15:01~C*01:02	0.0023	1	1.0000	<0.0001	2.0
B*35:01~C*03:04	0.0023	1	−0.9635	<0.0001	−10.1
B*35:14~C*04:01	0.0023	1	1.0000	0.0305	2.0
B*39:39~C*01:02	0.0023	1	1.0000	<0.0001	2.0
B*40:02~C*04:01	0.0023	1	−0.9531	<0.0001	−9.2
B*42:02~C*17:01	0.0023	1	1.0000	<0.0001	2.0
B*44:02~C*05:01	0.0023	1	1.0000	<0.0001	2.0
B*51:01~C*08:01	0.0023	1	1.0000	<0.0001	2.0
B*51:01~C*15:02	0.0023	1	0.2168	0.0385	1.7
B*52:01~C*12:02	0.0023	1	1.0000	<0.0001	2.0
B*52:01~C*15:15	0.0023	1	1.0000	<0.0001	2.0
Native American blocks ABF:	0.8441				
European blocks ABF:	0.0590				
African blocks ABF:	0.0138				
Asian blocks ABF:	0.0046				
Not previously reported blocks ABF:	0.0782				

*Only haplotypes found more than once (H.F.: Haplotype frequency ≥ 0.0046) were included in the table. ABF: Aggregate block frequency.

**Table 4:** HLA class II (*HLA-DRB1*~*DRB3|4|5*~*DQB1*~*DQA1*~*DPA1*~*DPB1*) haplotypes* in 218 Lacandon Native Americans.

HLA~DRB1~DRB3/4/5~DQB1~DQA1~DPA1~DPB1 haplotype	H.F.	n (N = 218)	Δ′	*p*	*t*
DRB1*04:11~DRB4*01:03~DQB1*03:02~DQA1*03:01~DPA1*01:03~DPB1*04:02	0.2047	89	0.6566	<0.0001	4.6
DRB1*04:07~DRB4*01:01~DQB1*03:02~DQA1*03:01~DPA1*01:03~DPB1*04:02	0.1449	63	0.6379	0.0012	3.8
DRB1*04:11~DRB4*01:01~DQB1*03:02~DQA1*03:01~DPA1*01:03~DPB1*04:02	0.1196	52	0.1168	0.5642	0.5
DRB1*16:02~DRB5*02:02~DQB1*03:01~DQA1*05:05~DPA1*01:03~DPB1*04:02	0.0575	25	0.5539	0.0890	2.0
DRB1*08:02~NULL~DQB1*04:02~DQA1*04:01~DPA1*01:03~DPB1*04:02	0.0552	24	0.5374	0.1056	1.9
DRB1*04:07~DRB4*01:03~DQB1*03:02~DQA1*03:01~DPA1*01:03~DPB1*04:02	0.0529	23	−0.0541	0.5728	−0.5
DRB1*14:02~DRB3*01:01~DQB1*03:01~DQA1*05:03~DPA1*01:03~DPB1*04:02	0.0414	18	−0.0129	0.9079	−0.1
DRB1*04:04~DRB4*01:03~DQB1*03:02~DQA1*03:01~DPA1*01:03~DPB1*04:02	0.0276	12	−0.0129	0.9254	−0.1
DRB1*14:06~DRB3*01:01~DQB1*03:01~DQA1*05:03~DPA1*01:03~DPB1*04:01	0.0253	11	0.8361	<0.0001	6.7
DRB1*04:04~DRB4*01:01~DQB1*03:02~DQA1*03:01~DPA1*02:02~DPB1*05:01	0.0230	10	0.6024	<0.0001	6.3
DRB1*11:04~DRB3*02:02~DQB1*03:01~DQA1*05:05~DPA1*01:03~DPB1*04:02	0.0184	8	−0.1901	0.2162	−1.0
DRB1*04:04~DRB4*01:01~DQB1*03:02~DQA1*03:01~DPA1*01:03~DPB1*04:02	0.0138	6	−0.5065	0.0002	−2.5
DRB1*04:03~DRB4*01:03~DQB1*03:02~DQA1*03:01~DPA1*01:03~DPB1*04:02	0.0115	5	1.0000	0.2062	2.1
DRB1*04:07~DRB4*01:03~DQB1*03:02~DQA1*03:01~DPA1*02:02~DPB1*05:01	0.0092	4	0.0903	0.0837	2.4
DRB1*04:11~DRB4*01:01~DQB1*03:02~DQA1*03:01~DPA1*01:03~DPB1*02:01	0.0092	4	0.1065	0.2753	1.7
DRB1*04:11~DRB4*01:03~DQB1*03:02~DQA1*03:01~DPA1*01:03~DPB1*02:01	0.0092	4	0.0298	0.8090	0.4
DRB1*04:03~DRB4*01:01~DQB1*03:02~DQA1*03:01~DPA1*01:03~DPB1*04:02	0.0069	3	1.0000	0.3286	1.6
DRB1*04:04~DRB4*01:03~DQB1*03:02~DQA1*03:01~DPA1*02:02~DPB1*05:01	0.0069	3	0.1386	0.0214	2.6
DRB1*04:11~DRB4*01:01~DQB1*03:02~DQA1*03:01~DPA1*01:03~DPB1*04:01	0.0069	3	−0.2565	0.5655	−1.1
DRB1*04:11~DRB4*01:03~DQB1*03:02~DQA1*03:01~DPA1*02:02~DPB1*05:01	0.0069	3	−0.4552	0.2154	−2.4
DRB1*08:01~NULL~DQB1*04:02~DQA1*04:01~DPA1*01:03~DPB1*04:01	0.0069	3	0.7337	<0.0001	3.4
DRB1*01:01~NULL~DQB1*05:01~DQA1*01:01~DPA1*01:03~DPB1*04:01	0.0046	2	1.0000	<0.0001	2.8
DRB1*04:07~DRB4*01:01~DQB1*03:02~DQA1*03:01~DPA1*01:03~DPB1*03:01	0.0046	2	0.1590	0.3141	1.5
DRB1*04:07~DRB4*01:05~DQB1*03:02~DQA1*03:01~DPA1*01:03~DPB1*04:02	0.0046	2	1.0000	0.4255	1.3
DRB1*04:11~DRB4*01:01~DQB1*03:02~DQA1*03:01~DPA1*02:02~DPB1*05:01	0.0046	2	−0.4662	0.3152	−2.1
DRB1*11:04~DRB3*02:02~DQB1*03:01~DQA1*05:05~DPA1*02:02~DPB1*05:01	0.0046	2	0.1029	0.1249	1.9
DRB1*13:03~DRB3*02:02~DQB1*02:02~DQA1*02:01~DPA1*02:02~DPB1*11:01	0.0046	2	1.0000	<0.0001	2.8
DRB1*16:02~DRB5*02:02~DQB1*03:01~DQA1*05:05~DPA1*01:03~DPB1*02:01	0.0046	2	0.0602	0.3216	1.4
Native American MPA blocks ABF:	0.6854				
European MPA blocks ABF:	0.1196				
African MPA blocks ABF:	0.0138				
Asian MPA blocks ABF:	0.0184				
Not previously reported blocks ABF:	0.1628				

*Only haplotypes found more than once (H.F.: Haplotype frequency ≥ 0.0046) were included. ABF: Aggregate block frequency.

**Table 5:** HLA Conserved Extended Haplotypes* (CEH) in 218 Lacandon Native Americans.

Conserved extended haplotype (CEH)	H.F.	n (N = 218)	Δ′	*p*	*t*
B*40:02~C*03:04~DRB1*04:11~DQB1*03:02	0.1702	74	0.6663	<0.0001	15.4
B*35:12~C*04:01~DRB1*04:11~DQB1*03:02	0.0759	33	0.5328	<0.0001	7.9
B*39:05~C*07:02~DRB1*04:07~DQB1*03:02	0.0759	33	0.2992	<0.0001	7.4
B*35:01~C*07:02~DRB1*04:11~DQB1*03:02	0.0575	25	−0.0679	0.6306	−0.7
B*35:01~C*07:02~DRB1*16:02~DQB1*03:01	0.0483	21	0.6710	<0.0001	8.9
B*40:08~C*03:04~DRB1*04:07~DQB1*03:02	0.0460	20	0.5985	<0.0001	7.8
B*39:05~C*07:02~DRB1*04:11~DQB1*03:02	0.0414	18	−0.3102	0.0306	−3.1
B*35:01~C*07:02~DRB1*04:07~DQB1*03:02	0.0368	16	−0.0478	0.8076	−0.4
B*40:02~C*03:04~DRB1*14:02~DQB1*03:01	0.0345	15	0.4677	<0.0001	6.2
B*35:01~C*04:01~DRB1*04:04~DQB1*03:02	0.0322	14	0.6416	<0.0001	7.5
B*40:02~C*15:02~DRB1*14:06~DQB1*03:01	0.0322	14	0.8699	<0.0001	7.7
B*18:01~C*07:01~DRB1*11:04~DQB1*03:01	0.0253	11	0.8417	<0.0001	6.8
B*39:05~C*07:02~DRB1*16:02~DQB1*03:01	0.0161	7	0.0999	0.1982	2.0
B*35:12~C*04:01~DRB1*04:07~DQB1*03:02	0.0138	6	−0.4378	0.0859	−3.1
B*39:05~C*07:02~DRB1*08:02~DQB1*04:02	0.0115	5	0.0332	0.6805	0.7
B*40:02~C*03:05~DRB1*04:07~DQB1*03:02	0.0115	5	0.5148	0.0067	3.5
B*40:08~C*03:04~DRB1*04:03~DQB1*03:02	0.0115	5	0.5255	<0.0001	4.3
B*35:12~C*04:01~DRB1*08:02~DQB1*04:02	0.0092	4	0.0507	0.4216	1.3
B*35:43~C*01:02~DRB1*04:07~DQB1*03:02	0.0092	4	0.7412	0.0021	3.6
B*39:08~C*07:02~DRB1*04:07~DQB1*03:02	0.0092	4	0.5687	0.0097	3.3
B*52:01~C*03:03~DRB1*04:04~DQB1*03:02	0.0092	4	0.7850	<0.0001	4.0
B*35:01~C*04:01~DRB1*08:02~DQB1*04:02	0.0069	3	0.0892	0.0947	2.2
B*35:01~C*07:02~DRB1*04:03~DQB1*03:02	0.0069	3	0.2049	0.1574	2.0
B*39:01~C*07:02~DRB1*04:11~DQB1*03:02	0.0069	3	0.2158	0.4805	1.1
B*39:05~C*07:02~DRB1*14:02~DQB1*03:01	0.0069	3	−0.2660	0.5464	−1.2
B*39:06~C*07:02~DRB1*04:04~DQB1*03:02	0.0069	3	0.4624	<0.0001	3.2
B*14:02~C*08:02~DRB1*01:01~DQB1*05:01	0.0046	2	1.0000	<0.0001	2.8
B*14:02~C*08:02~DRB1*01:02~DQB1*05:01	0.0046	2	0.6637	<0.0001	2.8
B*35:01~C*04:01~DRB1*04:07~DQB1*03:02	0.0046	2	−0.5806	0.1399	−2.9
B*35:01~C*04:01~DRB1*04:11~DQB1*03:02	0.0046	2	−0.7372	0.0091	−4.1
B*35:01~C*07:02~DRB1*04:04~DQB1*03:02	0.0046	2	−0.6132	0.1144	−3.4
B*35:01~C*07:02~DRB1*08:02~DQB1*04:02	0.0046	2	−0.5415	0.2029	−2.7
B*35:12~C*04:01~DRB1*14:02~DQB1*03:01	0.0046	2	−0.2504	−0.6566	−0.9
B*39:01~C*07:02~DRB1*04:07~DQB1*03:02	0.0046	2	0.1375	0.5317	1.0
B*40:02~C*15:02~DRB1*08:02~DQB1*04:02	0.0046	2	0.0554	0.3378	1.4
B*40:08~C*03:04~DRB1*04:11~DQB1*03:02	0.0046	2	−0.8097	0.0007	−5.3
B*40:11~C*03:04~DRB1*08:02~DQB1*04:02	0.0046	2	1.0000	<0.0001	2.8
B*51:01~C*15:04~DRB1*04:04~DQB1*03:02	0.0046	2	1.0000	<0.0001	2.8
B*53:01~C*04:01~DRB1*13:03~DQB1*02:02	0.0046	2	1.0000	<0.0001	2.8
Native American MPA CEH frequency:	0.3188				
European MPA CEH frequency:	0.0917				
African MPA CEH frequency:	0.0023				
Asian MPA CEH frequency:	0.0023				
Not previously reported CEH frequency:	0.6055				

*Only haplotypes found more than once (H.F.: Haplotype frequency ≥ 0.0046) were included.

**Table 6:** The extension of HLA Conserved Extended Haplotypes* (CEH) to *HLA*−*A* in 218 Lacandon Native Americans.

HLA~A~B~C~DRB1~DQB1 haplotype	H.F.	n (N = 218)	Δ′	*p*	*t*
A*31:01~B*40:02~C*03:04~DRB1*04:11~DQB1*03:02	0.1311	57	0.9216	0.0000	14.8
A*02:06~B*35:01~C*07:02~DRB1*04:11~DQB1*03:02	0.0483	21	0.4003	0.0013	4.7
A*24:02~B*35:12~C*04:01~DRB1*04:11~DQB1*03:02	0.0483	21	0.6079	0.0000	6.3
A*68:03~B*35:01~C*07:02~DRB1*16:02~DQB1*03:01	0.0483	21	0.7020	0.0000	9.1
A*68:01~B*40:08~C*03:04~DRB1*04:07~DQB1*03:02	0.0460	20	0.6303	0.0000	7.7
A*68:01~B*39:05~C*07:02~DRB1*04:11~DQB1*03:02	0.0414	18	0.5608	0.0001	5.5
A*68:03~B*39:05~C*07:02~DRB1*04:07~DQB1*03:02	0.0391	17	0.6226	0.0000	7.0
A*24:02~B*40:02~C*15:02~DRB1*14:06~DQB1*03:01	0.0322	14	1.0000	0.0000	7.5
A*02:01~B*18:01~C*07:01~DRB1*11:04~DQB1*03:01	0.0253	11	0.8417	0.0000	6.6
A*02:01~B*35:12~C*04:01~DRB1*04:11~DQB1*03:02	0.0230	10	0.7386	0.0006	4.9
A*24:14~B*35:01~C*04:01~DRB1*04:04~DQB1*03:02	0.0230	10	1.0000	0.0000	6.2
A*02:06~B*40:02~C*03:04~DRB1*04:11~DQB1*03:02	0.0207	9	0.5174	0.0121	3.6
A*24:14~B*40:02~C*03:04~DRB1*14:02~DQB1*03:01	0.0207	9	0.8072	0.0000	5.8
A*02:06~B*35:01~C*07:02~DRB1*04:07~DQB1*03:02	0.0161	7	−0.0933	0.7592	−0.5
A*24:02~B*39:05~C*07:02~DRB1*04:07~DQB1*03:02	0.0161	7	0.4029	0.0066	3.6
A*68:03~B*35:01~C*07:02~DRB1*04:07~DQB1*03:02	0.0161	7	−0.0933	0.7592	−0.5
A*68:01~B*40:08~C*03:04~DRB1*04:03~DQB1*03:02	0.0115	5	0.5266	0.0000	4.2
A*24:02~B*35:12~C*04:01~DRB1*04:07~DQB1*03:02	0.0092	4	−0.3709	0.2723	−2.0
A*68:05~B*39:05~C*07:02~DRB1*04:07~DQB1*03:02	0.0092	4	0.5687	0.0097	3.2
A*02:01~B*35:43~C*01:02~DRB1*04:07~DQB1*03:02	0.0069	3	1.0000	0.0013	3.3
A*02:06~B*40:02~C*03:04~DRB1*14:02~DQB1*03:01	0.0069	3	0.1845	0.0060	2.7
A*02:06~B*52:01~C*03:03~DRB1*04:04~DQB1*03:02	0.0069	3	1.0000	0.0000	3.4
A*24:02~B*39:06~C*07:02~DRB1*04:04~DQB1*03:02	0.0069	3	0.4624	0.0000	3.1
A*68:01~B*39:01~C*07:02~DRB1*04:11~DQB1*03:02	0.0069	3	0.6079	0.1053	2.3
A*68:01~B*39:05~C*07:02~DRB1*16:02~DQB1*03:01	0.0069	3	0.0605	0.2314	1.7
A*68:03~B*35:01~C*07:02~DRB1*04:11~DQB1*03:02	0.0069	3	−0.7566	0.0005	−5.2
A*02:01~B*35:12~C*04:01~DRB1*08:02~DQB1*04:02	0.0046	2	0.1145	0.1084	1.9
A*02:06~B*35:01~C*04:01~DRB1*04:04~DQB1*03:02	0.0046	2	1.0000	0.0000	2.7
A*02:06~B*35:01~C*07:02~DRB1*04:03~DQB1*03:02	0.0046	2	0.1599	0.0872	1.9
A*02:06~B*35:01~C*07:02~DRB1*04:04~DQB1*03:02	0.0046	2	−0.1581	0.7929	−0.5
A*02:06~B*39:08~C*07:02~DRB1*04:07~DQB1*03:02	0.0046	2	0.5687	0.0682	2.2
A*02:06~B*40:02~C*15:02~DRB1*08:02~DQB1*04:02	0.0046	2	1.0000	0.0000	2.7
A*24:02~B*35:01~C*04:01~DRB1*04:04~DQB1*03:02	0.0046	2	0.3549	0.0036	2.4
A*24:02~B*35:01~C*04:01~DRB1*04:11~DQB1*03:02	0.0046	2	0.0589	0.8606	0.3
A*24:02~B*39:05~C*07:02~DRB1*08:02~DQB1*04:02	0.0046	2	0.1008	0.1406	1.8
A*24:02~B*40:02~C*03:04~DRB1*04:11~DQB1*03:02	0.0046	2	0.2158	0.5654	0.9
A*24:02~B*40:02~C*03:04~DRB1*14:02~DQB1*03:01	0.0046	2	0.4699	0.0001	2.6
A*24:02~B*40:02~C*03:05~DRB1*04:07~DQB1*03:02	0.0046	2	1.0000	0.0089	2.7
A*24:14~B*40:02~C*03:04~DRB1*04:11~DQB1*03:02	0.0046	2	−0.4984	0.2071	−2.0
A*33:01~B*14:02~C*08:02~DRB1*01:02~DQB1*05:01	0.0046	2	1.0000	0.0000	2.7
A*68:01~B*35:01~C*04:01~DRB1*04:07~DQB1*03:02	0.0046	2	1.0000	0.0089	2.7
A*68:01~B*39:05~C*07:02~DRB1*04:07~DQB1*03:02	0.0046	2	−0.6477	0.0710	−3.5
A*68:02~B*53:01~C*04:01~DRB1*13:03~DQB1*02:02	0.0046	2	1.0000	0.0000	2.7
A*68:03~B*39:01~C*07:02~DRB1*04:07~DQB1*03:02	0.0046	2	1.0000	0.0089	2.7
A*68:03~B*39:05~C*07:02~DRB1*08:02~DQB1*04:02	0.0046	2	0.0259	0.6024	0.8
A*68:03~B*39:05~C*07:02~DRB1*16:02~DQB1*03:01	0.0046	2	0.0214	0.6792	0.7
Native American MPA CEH + *HLA-A* frequency:	0.1092				
European MPA CEH + *HLA-A* frequency:	0.0480				
African MPA CEH + *HLA-A* frequency:	0.0066				
Asian MPA CEH + *HLA-A* frequency:	0.0349				
Not previously reported CEH + *HLA-A* frequency:	0.7663				

* Only haplotypes found more than once (H.F.: Haplotype frequency ≥ 0.0046) were included. MPA: Most probable ancestry. CEH: conserved extended haplotype.

**Table 7:** *HLA-A*~*B*~*C*~*DRB1*~*DRB3*/*4*/*5*~*DQB1*~*DQA1*~*DPA1*~*DPB1* haplotypes* in 218 Lacandon Native Americans.

HLA~A~B~C~DRB1~DRB3/4/5~DQB1~DQA1~DPA1~DPB1 haplotype	H.F.	n (N = 218)	Δ′	*p*	*t*
A*31:01~B*40:02~C*03:04~DRB1*04:11~DRB4*01:03~DQB1*03:02~DQA1*03:01~DPA1*01:03~DPB1*04:02	0.0667	29	0.3587	<0.0001	8.3
A*31:01~B*40:02~C*03:04~DRB1*04:11~DRB4*01:01~DQB1*03:02~DQA1*03:01~DPA1*01:03~DPB1*04:02	0.0529	23	0.3582	<0.0001	8.3
A*68:03~B*35:01~C*07:02~DRB1*16:02~DRB5*02:02~DQB1*03:01~DQA1*05:05~DPA1*01:03~DPB1*04:02	0.0414	18	0.6975	<0.0001	8.7
A*24:02~B*35:12~C*04:01~DRB1*04:11~DRB4*01:03~DQB1*03:02~DQA1*03:01~DPA1*01:03~DPB1*04:02	0.0368	16	0.4681	0.0000	6.6
A*68:01~B*39:05~C*07:02~DRB1*04:11~DRB4*01:03~DQB1*03:02~DQA1*03:01~DPA1*01:03~DPB1*04:02	0.0345	15	0.5035	<0.0001	6.5
A*02:06~B*35:01~C*07:02~DRB1*04:11~DRB4*01:03~DQB1*03:02~DQA1*03:01~DPA1*01:03~DPB1*04:02	0.0299	13	0.2334	0.0040	4.2
A*68:03~B*39:05~C*07:02~DRB1*04:07~DRB4*01:01~DQB1*03:02~DQA1*03:01~DPA1*01:03~DPB1*04:02	0.0299	13	0.4686	0.0000	6.4
A*24:02~B*40:02~C*15:02~DRB1*14:06~DRB3*01:01~DQB1*03:01~DQA1*05:03~DPA1*01:03~DPB1*04:01	0.0253	11	1.0000	<0.0001	6.8
A*68:01~B*40:08~C*03:04~DRB1*04:07~DRB4*01:03~DQB1*03:02~DQA1*03:01~DPA1*01:03~DPB1*04:02	0.0230	10	0.3980	<0.0001	6.0
A*02:01~B*18:01~C*07:01~DRB1*11:04~DRB3*02:02~DQB1*03:01~DQA1*05:05~DPA1*01:03~DPB1*04:02	0.0161	7	0.8713	<0.0001	5.4
A*02:01~B*35:12~C*04:01~DRB1*04:11~DRB4*01:03~DQB1*03:02~DQA1*03:01~DPA1*01:03~DPB1*04:02	0.0161	7	0.4828	0.0006	4.3
A*02:06~B*35:01~C*07:02~DRB1*04:11~DRB4*01:01~DQB1*03:02~DQA1*03:01~DPA1*01:03~DPB1*04:02	0.0161	7	0.1042	0.0777	2.6
A*68:01~B*40:08~C*03:04~DRB1*04:07~DRB4*01:01~DQB1*03:02~DQA1*03:01~DPA1*01:03~DPB1*04:02	0.0138	6	0.0890	0.2238	1.9
A*24:14~B*35:01~C*04:01~DRB1*04:04~DRB4*01:01~DQB1*03:02~DQA1*03:01~DPA1*02:02~DPB1*05:01	0.0115	5	0.4888	<0.0001	4.5
A*02:06~B*35:01~C*07:02~DRB1*04:07~DRB4*01:01~DQB1*03:02~DQA1*03:01~DPA1*01:03~DPB1*04:02	0.0092	4	−0.1447	0.7261	−0.7
A*24:14~B*40:02~C*03:04~DRB1*14:02~DRB3*01:01~DQB1*03:01~DQA1*05:03~DPA1*01:03~DPB1*04:02	0.0092	4	0.3376	<0.0001	3.8
A*68:03~B*35:01~C*07:02~DRB1*04:07~DRB4*01:01~DQB1*03:02~DQA1*03:01~DPA1*01:03~DPB1*04:02	0.0092	4	−0.1447	0.7261	−0.7
A*02:01~B*35:43~C*01:02~DRB1*04:07~DRB4*01:01~DQB1*03:02~DQA1*03:01~DPA1*01:03~DPB1*04:02	0.0069	3	1.0000	<0.0001	3.5
A*02:06~B*40:02~C*03:04~DRB1*04:11~DRB4*01:03~DQB1*03:02~DQA1*03:01~DPA1*01:03~DPB1*02:01	0.0069	3	0.7427	<0.0001	3.5
A*02:06~B*40:02~C*03:04~DRB1*14:02~DRB3*01:01~DQB1*03:01~DQA1*05:03~DPA1*01:03~DPB1*04:02	0.0069	3	0.1993	0.0003	3.1
A*02:06~B*52:01~C*03:03~DRB1*04:04~DRB4*01:03~DQB1*03:02~DQA1*03:01~DPA1*01:03~DPB1*04:02	0.0069	3	1.0000	<0.0001	3.5
A*24:02~B*35:12~C*04:01~DRB1*04:07~DRB4*01:01~DQB1*03:02~DQA1*03:01~DPA1*01:03~DPB1*04:02	0.0069	3	−0.2211	0.6297	−0.9
A*24:02~B*39:05~C*07:02~DRB1*04:07~DRB4*01:03~DQB1*03:02~DQA1*03:01~DPA1*01:03~DPB1*04:02	0.0069	3	0.1901	0.0025	2.9
A*24:02~B*39:06~C*07:02~DRB1*04:04~DRB4*01:03~DQB1*03:02~DQA1*03:01~DPA1*01:03~DPB1*04:02	0.0069	3	0.4865	<0.0001	3.4
A*68:01~B*39:01~C*07:02~DRB1*04:11~DRB4*01:01~DQB1*03:02~DQA1*03:01~DPA1*01:03~DPB1*04:02	0.0069	3	0.7180	0.0001	3.3
A*68:01~B*39:05~C*07:02~DRB1*04:11~DRB4*01:01~DQB1*03:02~DQA1*03:01~DPA1*01:03~DPB1*04:02	0.0069	3	0.0073	0.9166	0.2
A*68:01~B*39:05~C*07:02~DRB1*16:02~DRB5*02:02~DQB1*03:01~DQA1*05:05~DPA1*01:03~DPB1*04:02	0.0069	3	0.0692	0.1387	2.0
A*68:03~B*35:01~C*07:02~DRB1*04:07~DRB4*01:03~DQB1*03:02~DQA1*03:01~DPA1*02:02~DPB1*05:01	0.0069	3	0.7300	<0.0001	3.4
A*02:01~B*18:01~C*07:01~DRB1*11:04~DRB3*02:02~DQB1*03:01~DQA1*05:05~DPA1*02:02~DPB1*05:01	0.0046	2	1.0000	<0.0001	2.8
A*02:01~B*35:12~C*04:01~DRB1*04:11~DRB4*01:01~DQB1*03:02~DQA1*03:01~DPA1*01:03~DPB1*04:02	0.0046	2	0.0599	0.5566	0.9
A*02:01~B*35:12~C*04:01~DRB1*08:02~NULL~DQB1*04:02~DQA1*04:01~DPA1*01:03~DPB1*04:02	0.0046	2	0.1206	0.0719	2.1
A*02:06~B*35:01~C*07:02~DRB1*04:07~DRB4*01:03~DQB1*03:02~DQA1*03:01~DPA1*01:03~DPB1*04:02	0.0046	2	0.0137	0.8113	0.4
A*02:06~B*39:08~C*07:02~DRB1*04:07~DRB4*01:01~DQB1*03:02~DQA1*03:01~DPA1*01:03~DPB1*04:02	0.0046	2	0.6135	0.0076	2.6
A*02:06~B*40:02~C*03:04~DRB1*04:11~DRB4*01:01~DQB1*03:02~DQA1*03:01~DPA1*01:03~DPB1*02:01	0.0046	2	0.4854	<0.0001	2.8
A*02:06~B*40:02~C*03:04~DRB1*04:11~DRB4*01:01~DQB1*03:02~DQA1*03:01~DPA1*01:03~DPB1*04:02	0.0046	2	0.0455	0.6421	0.8
A*24:02~B*35:12~C*04:01~DRB1*04:11~DRB4*01:01~DQB1*03:02~DQA1*03:01~DPA1*01:03~DPB1*04:02	0.0046	2	−0.3709	0.4685	−1.5
A*24:02~B*39:05~C*07:02~DRB1*04:07~DRB4*01:01~DQB1*03:02~DQA1*03:01~DPA1*01:03~DPB1*04:02	0.0046	2	0.0189	0.8626	0.3
A*24:02~B*39:05~C*07:02~DRB1*08:02~NULL~DQB1*04:02~DQA1*04:01~DPA1*01:03~DPB1*04:02	0.0046	2	0.1071	0.0959	2.0
A*24:02~B*40:02~C*03:04~DRB1*14:02~DRB3*01:01~DQB1*03:01~DQA1*05:03~DPA1*01:03~DPB1*04:02	0.0046	2	0.4795	<0.0001	2.7
A*24:14~B*35:01~C*04:01~DRB1*04:04~DRB4*01:01~DQB1*03:02~DQA1*03:01~DPA1*01:03~DPB1*04:02	0.0046	2	0.3185	<0.0001	2.7
A*24:14~B*35:01~C*04:01~DRB1*04:04~DRB4*01:03~DQB1*03:02~DQA1*03:01~DPA1*02:02~DPB1*05:01	0.0046	2	0.6592	<0.0001	2.8
A*24:14~B*40:02~C*03:04~DRB1*04:11~DRB4*01:01~DQB1*03:02~DQA1*03:01~DPA1*01:03~DPB1*04:02	0.0046	2	0.0770	0.4699	1.1
A*31:01~B*40:02~C*03:04~DRB1*04:11~DRB4*01:01~DQB1*03:02~DQA1*03:01~DPA1*02:02~DPB1*05:01	0.0046	2	1.0000	0.0003	2.8
A*68:01~B*40:08~C*03:04~DRB1*04:03~DRB4*01:01~DQB1*03:02~DQA1*03:01~DPA1*01:03~DPB1*04:02	0.0046	2	0.6450	<0.0001	2.7
A*68:01~B*40:08~C*03:04~DRB1*04:03~DRB4*01:03~DQB1*03:02~DQA1*03:01~DPA1*01:03~DPB1*04:02	0.0046	2	0.3609	0.0015	2.6
A*68:01~B*40:08~C*03:04~DRB1*04:07~DRB4*01:05~DQB1*03:02~DQA1*03:01~DPA1*01:03~DPB1*04:02	0.0046	2	1.0000	<0.0001	2.8
A*68:02~B*53:01~C*04:01~DRB1*13:03~DRB3*02:02~DQB1*02:02~DQA1*02:01~DPA1*02:02~DPB1*11:01	0.0046	2	1.0000	<0.0001	2.8
A*68:03~B*35:01~C*07:02~DRB1*04:11~DRB4*01:03~DQB1*03:02~DQA1*03:01~DPA1*01:03~DPB1*04:02	0.0046	2	−0.6973	0.0380	−4.3
A*68:03~B*35:01~C*07:02~DRB1*16:02~DRB5*02:02~DQB1*03:01~DQA1*05:05~DPA1*01:03~DPB1*02:01	0.0046	2	1.0000	<0.0001	2.8
A*68:03~B*39:01~C*07:02~DRB1*04:07~DRB4*01:01~DQB1*03:02~DQA1*03:01~DPA1*01:03~DPB1*04:02	0.0046	2	1.0000	0.0004	2.8
A*68:03~B*39:05~C*07:02~DRB1*04:07~DRB4*01:03~DQB1*03:02~DQA1*03:01~DPA1*01:03~DPB1*04:02	0.0046	2	0.0365	0.4454	1.2
A*68:03~B*39:05~C*07:02~DRB1*08:02~NULL~DQB1*04:02~DQA1*04:01~DPA1*01:03~DPB1*04:02	0.0046	2	0.0326	0.4848	1.1
A*68:03~B*39:05~C*07:02~DRB1*16:02~DRB5*02:02~DQB1*03:01~DQA1*05:05~DPA1*01:03~DPB1*04:02	0.0046	2	0.0304	0.5242	1.0
A*68:05~B*39:05~C*07:02~DRB1*04:07~DRB4*01:01~DQB1*03:02~DQA1*03:01~DPA1*01:03~DPB1*04:02	0.0046	2	0.2270	0.1611	1.8

* Only haplotypes found more than once (H.F.: Haplotype frequency ≥ 0.0046) were included.

### Diversity of Lacandon HLA alleles

Forensic parameters of genetic diversity were calculated to assess HLA diversity in Lacandon using polymorphism information content (PIC), power of discrimination (PD), and Hardy-Weinberg equilibrium (HWE) (Table 8). In this regard, *HLA-A*, *HLA-B* and *HLA-DRB1* were the most polymorphic loci with PIC values of 0.8444, 0.8227 and 0.7555 respectively; whereas *HLA-DPB1* and *HLA-DPA1* were the less diverse HLA loci with PIC values of 0.3547 and 0.1581, respectively. A significantly (*p* < 0.05) lower observed heterozygosity (OH) than expected heterozygosity (EH) was observed for *HLA-B*, *HLA-C*, *HLA-DRB1*, *HLA-DRB3*/4/5, *HLA-DQA1*, and *HLA-DQB1* loci. In contrast, *HLA-A* locus exhibited a higher OH than EH value (*p* < 0.0001).

**Table 8:** Calculated parameters of genetic diversity at the allele level for HLA system in a sample of Lacandon individuals.

Gene	O. H.	E. H.	*p* value	PIC	PD
*HLA-A*	0.8739	0.8623	<0.0001	0.8444	0.9565
*HLA-B*	0.8018	0.8419	0.0001	0.8227	0.9417
*HLA-C*	0.6622	0.7410	<0.0001	0.6993	0.8846
*HLA-DRB1*	0.7149	0.7813	<0.0001	0.7555	0.9255
*HLA-DRB3*/*4*/*5*	0.4781	0.4938	0.0479	0.4692	0.7129
*HLA-DQA1*	0.4670	0.4773	0.0003	0.4521	0.6894
*HLA-DQB1*	0.4493	0.4698	0.0001	0.4257	0.6559
*HLA-DPA1*	0.1659	0.1665	0.0967	0.1581	0.2943
*HLA-DPB1*	0.3750	0.3672	0.1866	0.3547	0.5881

O.H.: Observed heterozygosity. E.H.: Expected Heterozygosity. PIC: Polymorphism Information Contents. PD: Power of Discrimination. p values < 0.05 are considered statistically significant and thus reflect differences between O.H. and E.H.

### Genetic similarities with other populations

**Figure 2:**
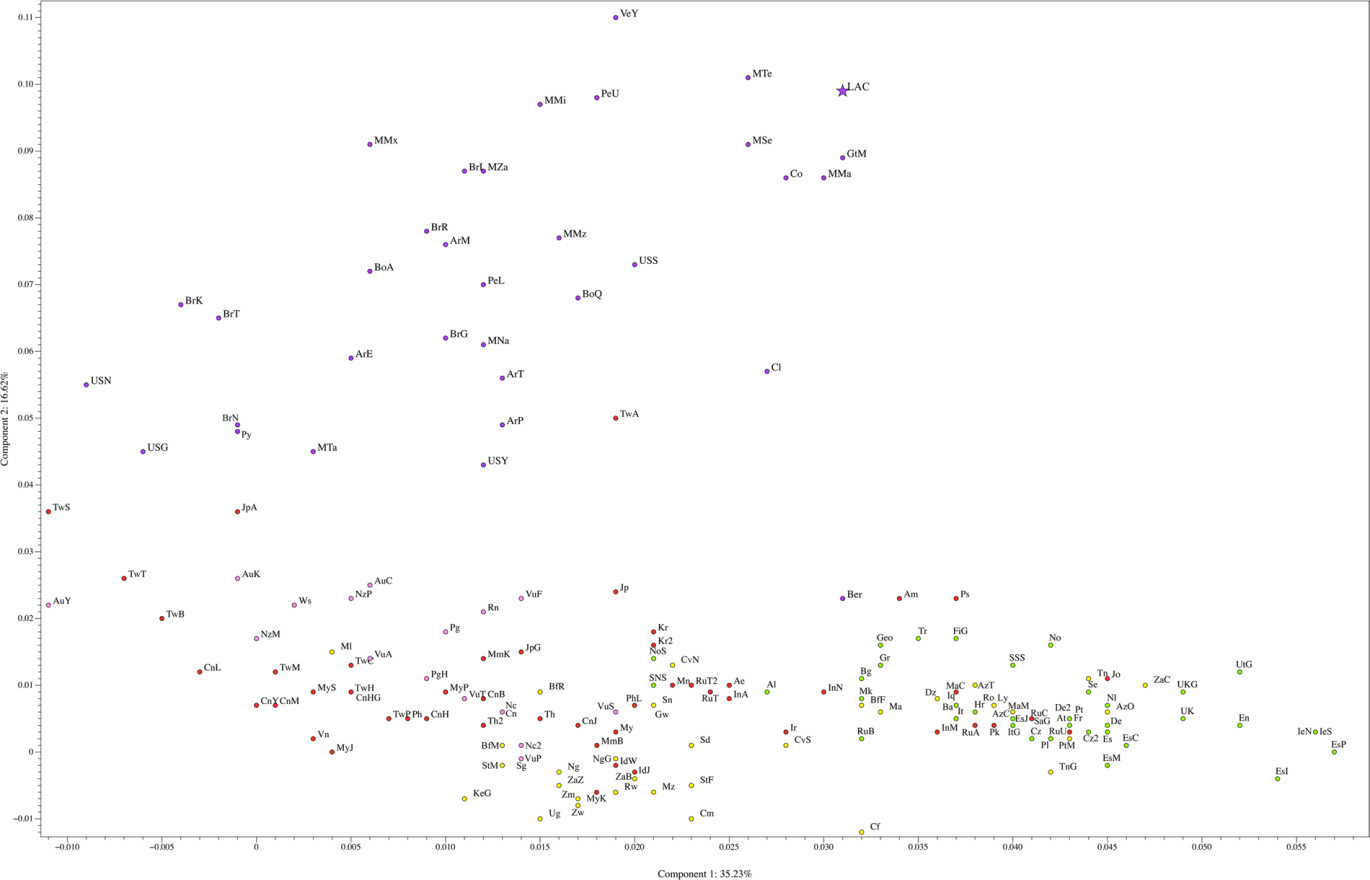
Principal Component Analysis for 163 populations (including the Lacandon group studied in this work). European populations are represented by green dots; African human groups correspond to yellow dots; red dots were assigned to Asian populations; Native American populations are represented by purple dots; Populations from Oceania are indicated with pink dots. Our Lacandon sample is represented by a purple star. For a view of the complete PCA, please refer to Supplementary Fig. 1. Argentina Gran Chaco Eastern Toba (n = 135) ArE; Argentina Gran Chaco Mataco Wichi (n = 49) ArM; Argentina Gran Chaco Western Toba Pilaga (n = 19) ArP; Argentina Rosario Toba (n = 86) ArT; Bolivia Aymara (n = 102) BoA; Bolivia Quechua (n = 80) BoQ; Brazil Ivaí Kaingang (n = 127) BrI; Brazil Río das cobras Guaraní (n = 98) BrG; Brazil Río das cobras Kaingang (n = 113) BrR; Brazil Terena (n = 60) BrT; Guatemala Maya (n = 132) GtM; Mexico Tarahumara (n = 44) MTa; Mexico Teenek (n = 55) MTe; Mexico Mayos (n = 60) MMa; Mexico Mazatecan (n = 89) MMz; Mexico Mixe (n = 55) MMx; Mexico Mixtec (n = 103) MMi; Mexico Zapotec (n = 90) MZa; Mexico Nahuas (n = 85) MNa; Mexico Seri (n = 34) MSe; Paraguay Guarani (n = 40) Py; Peru Lama (n = 83) PeL; Peru Titikaka Lake Uro (n = 105) PeU; USA Gila River (n = 492) USG; USA Sioux (n = 302) USS; USA Yupik (n = 252) USY; Venezuela Yucpa (n = 73) VeY. The complete list of abbreviations is included in Supplementary Table 1. Component 1 bears 35.23% of the total variance, while component 2 is explained by 16.62% of the variance. For the zoom-in of this PCA, please see Supplementary Fig. 1.

**Figure 3:**
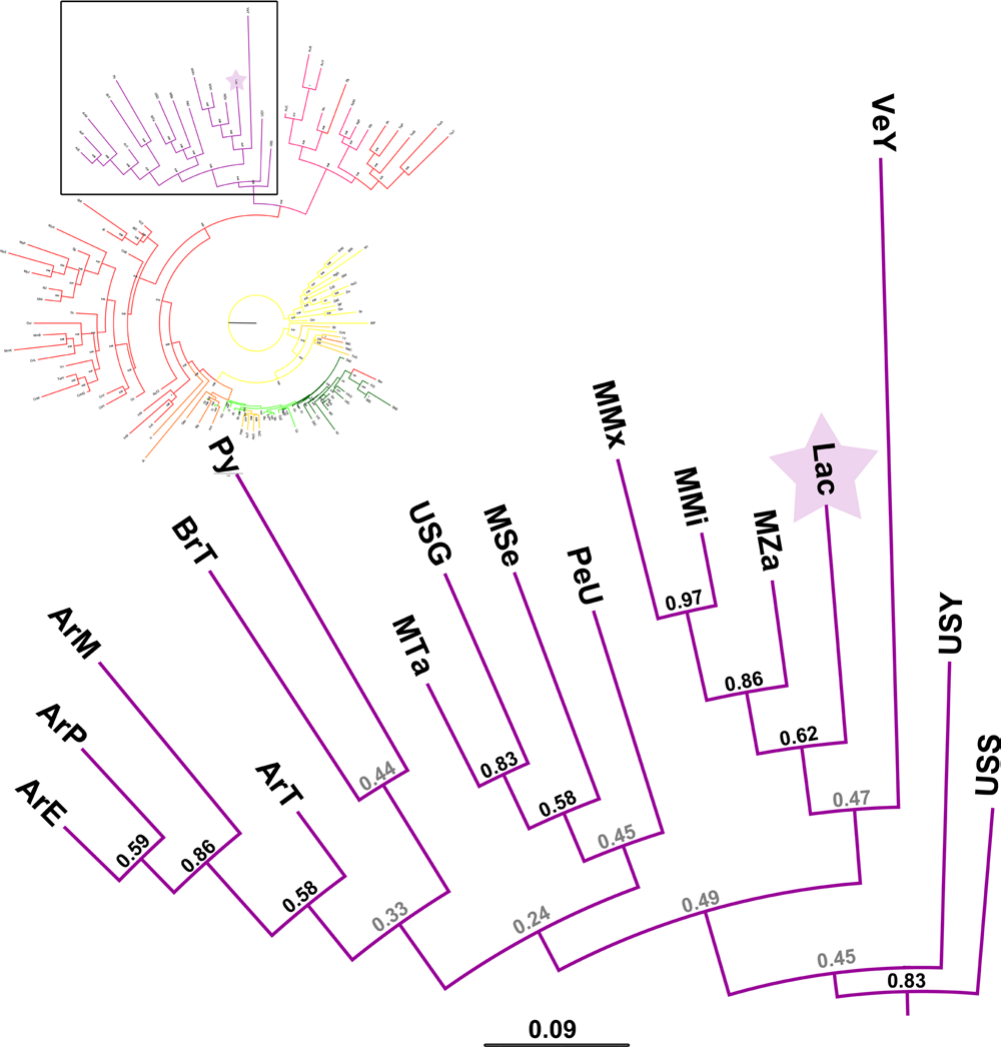
Zoom-in to the Native American cluster of the population phylogenetic tree built with 114 human groups (including Lacandon Mayan from this work). European populations are represented by green branches; African human groups correspond to yellow branches; red branches were assigned to Asian populations; Native American populations are represented by purple branches; populations from Oceania are indicated with pink branches. Our Lacandon sample is highlighted with a purple star. For a view of the complete tree, please refer to Supplementary Fig. 2. Argentina Gran Chaco Eastern Toba (n = 135) ArE; Argentina Gran Chaco Mataco Wichi (n = 49) ArM; Argentina Gran Chaco Western Toba Pilaga (n = 19) ArP; Argentina Rosario Toba (n = 86) ArT; Brazil Terena (n = 60) BrT; Mexico Lacandon (n = 228) Lac; Mexico Mixe (n = 55) MMx; Mexico Mixtec (n = 103) MMi; Mexico Seri (n = 34) MSe; Mexico Tarahumara (n = 44) MTa; Mexico Zapotec (n = 90) MZa; Paraguay Guarani (n = 40) Py; Peru Titikaka Lake Uro (n = 105) PeU; USA Gila River (n = 492) USG; USA Sioux (n = 302) USS; USA Yupik (n = 252) USY; Venezuela Yucpa (n = 73) VeY. References for all populations of this dataset are listed in Supplementary Table 1. For the zoom-out of this phylogenetic tree, please see Supplementary Fig. 2.

A PCA plot and a population phylogenetic tree were constructed using 180 populations (including the Lacandon group studied in this work) with *HLA-A*, *HLA-B* and *HLA-DRB1* data from a worldwide population dataset. Figure 2 and Supplementary Fig. 1 illustrate the results of the Principal Components Analysis (PCA). The Lacandon Mayans (purple star) cluster together with other Mexican Native American and Mexican Admixed populations, including Mayans from Guatemala. In addition, a Neighbor-Joining (NJ) analysis (Fig. 3**)** revealed that Lacandon Mayans are more closely related to Mixe, Mixtec and Zapotec Mexican Native American populations, which are geographically speaking the closest ones to the Lacandon Mayans. It is interesting to note that using HLA genes as genetic estimators, it is possible to mimic the results (although not to the same resolution) obtained with genome-wide data^49^.

### Non-overlapping associations between HLA alleles

**Figure 4:**
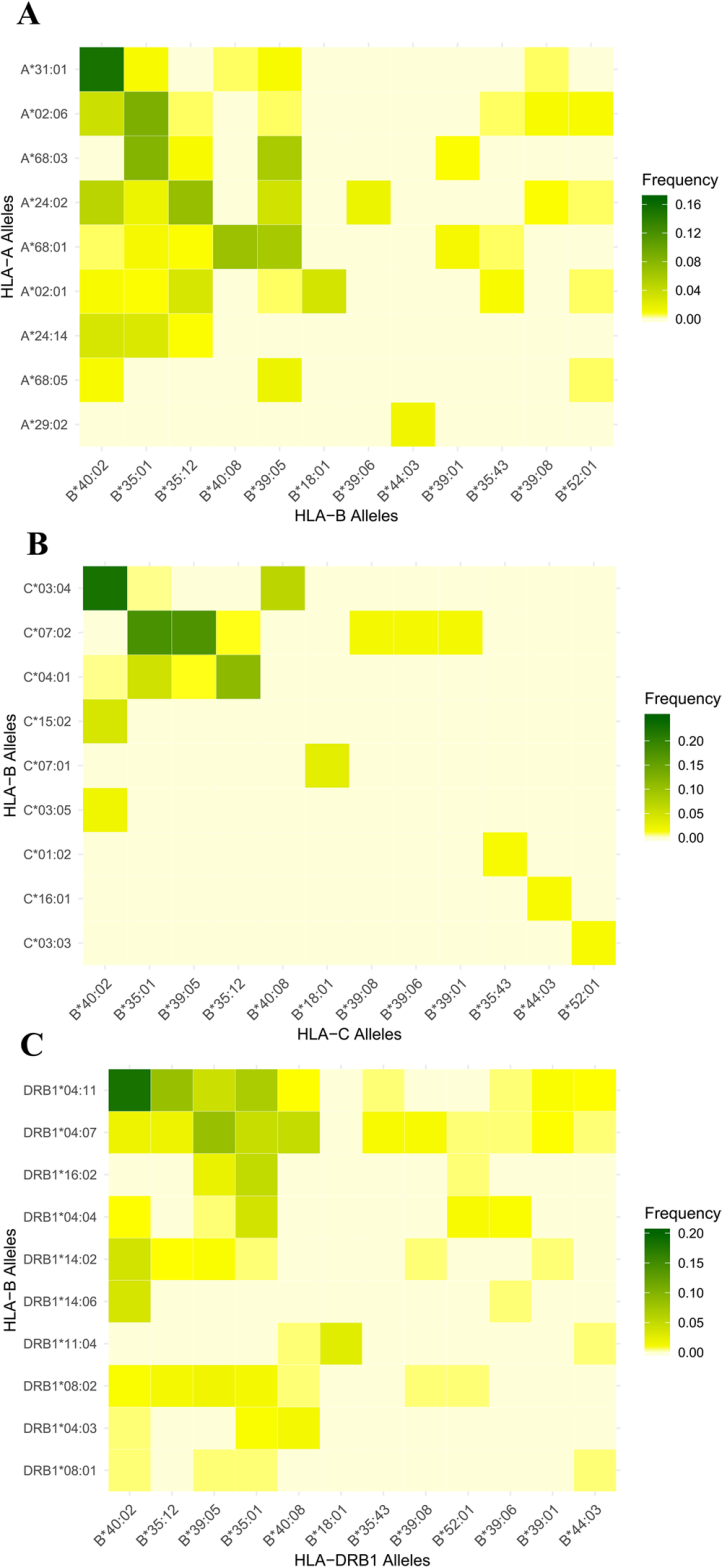
HLA associations in the Lacandon Maya sample from SE Mexico. (**A**) *HLA-A*~*B* associations, (**B**) *HLA-B*~*C* associations and (**C**) *HLA-B*~*DRB1* associations.

**Figure 5:**
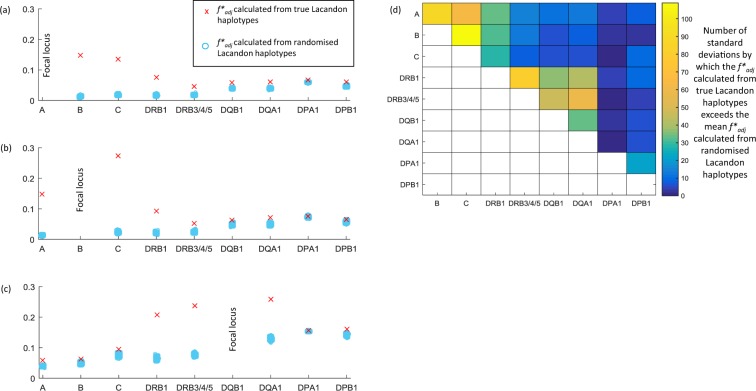
HLA ${f}_{adj}^{\ast }$ scores. The red crosses in panels (A–C) illustrate non-overlap measured using ${f}_{adj}^{\ast }$ for (**A**) *HLA-A* and all other HLA loci in the Lacandon dataset; (**B**) *HLA-B* and all other HLA loci in the dataset and (**C**) *HLA-DQB1* and all other HLA loci in the dataset. For each pair of loci we also generated 5000 different random combinations of the alleles found at each locus, and calculated ${f}_{adj}^{\ast }$ scores obtained from those randomized combinations. The ${f}_{adj}^{\ast }$ scores from the random combinations are shown as blue markers. These individual markers overlay one another at the scale used here, but illustrate the distribution of randomized scores. The heatmap in panel (**D**) indicates by how many standard deviations of the randomized ${f}_{adj}^{\ast }$ score distribution the actual ${f}_{adj}^{\ast }$ for a pair of loci in the Lacandon dataset exceeds the mean of the randomized ${f}_{adj}^{\ast }$ distribution for that pair.

Plots of the frequencies of all possible *HLA-A*~*B*, *HLA-B*~*C* and *HLA-B*~*DRB1* allele combinations are shown in Fig. 4, and plots for all HLA class I and class II associations can be found in Supplementary Fig. 3. Visual inspection suggests a high degree of non-overlap between *HLA-B* and *-C* in particular. Figure 5 displays the ${f}_{adj}^{\ast }$ metric (a parameter used to rank the strength of non-overlapping associations between different pairs of HLA loci) for all possible pairwise combinations of HLA loci with *HLA-A* **(4a)**, with *HLA-B* (**4b**) and with *HLA-DRB1* (**4c**), showing also the distribution of ${f}_{adj}^{\ast }$ values obtained when the alleles at the relevant loci are randomized (retaining their population frequencies within the dataset). The heatmap in Fig. 4 **(4d)** illustrates how many standard deviations above the mean randomized ${f}_{adj}^{\ast }$ value the actual Lacandon ${f}_{adj}^{\ast }$ value is for each indicated pair of loci. *HLA-B* and *-C* exhibit the highest degree of non-overlap by this measure, followed by *HLA-A* and *-B*, *HLA-DRB1* and *HLA-DRB3/*4*/*5, *HLA-A* and *-C*, and *HLA-DRB3/*4*/*5 and *HLA-DQA1*. All ${f}_{adj}^{\ast }$ values for each pair of HLA loci in the dataset are displayed in Supplementary Table 4.

### Assessment of the correlation between pathogen richness and HLA diversity

**Figure 6:**
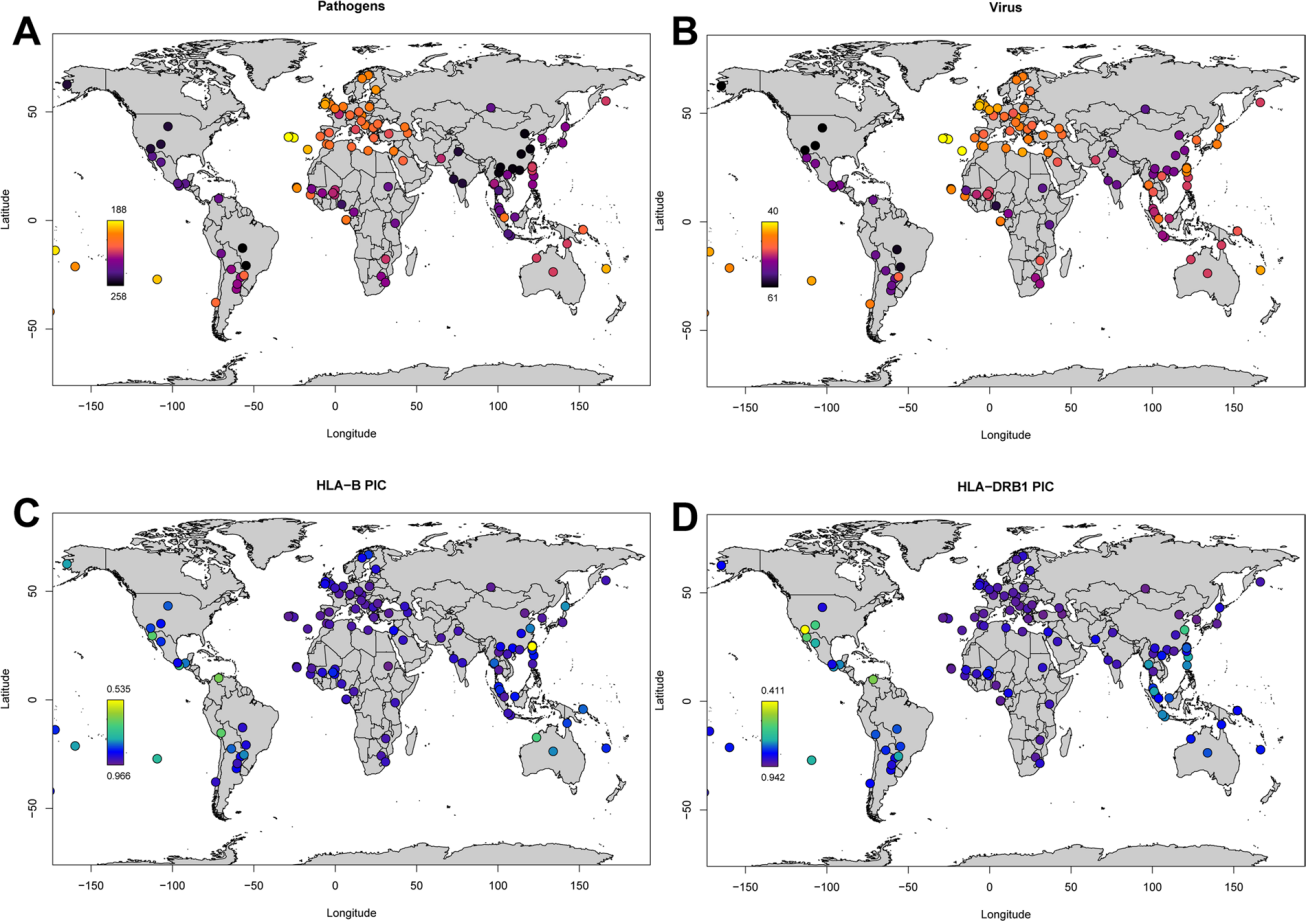
Worldwide distribution of pathogen and viral richness and genetic diversity for HLA. (**A**) Pathogen richness; (**B**) Viral richness; (**C**) *HLA-B* genetic diversity as estimated with polymorphism informative content (PIC) as a proxy for HLA class I diversity; *HLA-DRB1* genetic diversity as estimated with PIC as a proxy for HLA class II diversity.

We calculated PIC values for 122 populations as an estimator of genetic diversity for *HLA-A*, *HLA-B* and *HLA- DRB1* high resolution data (see Supplementary Table 1). We extracted pathogen and viral richness data from the GIDEON database^57^. We first tested the correlation between genetic diversity for each gene and geographic distance from Africa. Distance was calculated from East Africa to the location of each sample set analyzed. The general outlooks for (a) pathogen richness; (b) viral richness; (c) HLA class I (represented by its most polymorphic gene, *HLA-B*) genetic diversity; and (d) HLA class II (represented by its most polymorphic gene, *HLA-DRB1*) genetic diversity are shown in Fig. 6. We ran linear regressions for the PIC values for all three HLA loci vs. the geographic distance from Africa (Supplementary Fig. 4). We found a similar tendency for the three genes analyzed: a general decrease in diversity when distance from Africa increases (*HLA-A*: r^2^ = 0.5444; *HLA-B*: r^2^ = 0.2787; *HLA-DRB1*: r^2^ = 0.3352; all these r^2^ values correspond to regressions ran after removing outliers). We then ran general linear models including both distance from Africa and either pathogen richness (Supplementary Table 3) or viral richness (Supplementary Table 4**)** as predictor variables. No relationships were apparent between *HLA-A* or *HLA-B* diversity and either pathogen or viral richness. The 95% confidence interval for the gradient of the relationship between *HLA-DRB1* diversity and both pathogen and viral richness suggests a negative relationship (Supplementary Tables 3 and 4), but these relationships do not retain significance following a Bonferroni correction. Furthermore, only the negative relationship between viral richness and *HLA-DRB1* diversity in the non-Native American populations retains a 95% confidence interval < 0 in the analysis excluding outliers.

## Discussion

In this work, we used next generation sequencing to carry out high resolution typing of the *HLA-A* to *HLA-DPB1* loci in a group of Lacandon Maya settled in the Lacandon Rainforest in the lowlands of Chiapas State in the southeast of Mexico. We determined the distribution of HLA alleles and CEHs and their possible ancestral origin, and assessed genetic diversity within the classical HLA genes. We also put together this Lacandon Maya population with other populations, both Native American and non-Native American, to asses not only genetic relationships but also their general tendency when correlating the genetic diversity of these populations with the geographic distance from Africa and both pathogen and viral richness.

The PCA plot (Fig. 2) showed that the Lacandon population is genetically similar to other Native American populations, such as Mayos^28^, Teenek^58^, Seri^25^, Maya^30^, Wayu^33^, and Quechua^34^. When we performed a phylogenetic analysis of the relationship between the Lacandon and other North and South American Native populations, we found that the Lacandon belonged to the same clade as Native North Americans from Oaxaca (Mixe, Mixtec and Zapotecs)^29^ and the very divergent Yucpa from Venezuela^35^. However, these analyses were performed using allelic frequencies, not haplotypic data. When it comes to HLA, allelic diversity in Mesoamerican-descent groups tends to group together most of the Native North American populations. Haplotypic diversity can distinguish finer scale relationships among Native American populations, reflecting that although a limited allelic diversity came into the continent when the first human settlers arrived, recombination played an important role in adaptation to new environments and population differentiation. This is exemplified by the fact that four out of the top ten CEH in the Lacandon were not previously reported (accounting for 30.58% of the total CEHs), although all four haplotypes contained alleles for both class I and class II that are commonly present in other Native American populations.

As it has been shown before^49^, Lacandon Mayans and other Mayan groups have a genetic component very distinct from other populations, even from those in the same (or at least related) linguistic families, or neighboring populations. This uniqueness can also be detected in the HLA genomic region. Distinctive alleles present in the Lacandon population include A*24:14 (A.F. = 0.0502; also found in the Mayan population from Guatemala with an A.F. = 0.0040^30^, Buryat from Mongolia with an A.F. = 0.0040^59^, and Saudi Arabia with and A.F. = 0.0032^60^) and B*40:08 [A.F. = 0.0611; also found in Texas Hispanic from USA with an A.F. = 0.0170 (data collected by Cathi Murphey, available in^61^)], Polynesian from American Samoa with an A.F. = 0.0100^62^ and northern India with an A.F. = 0.0100^63^. Furthermore, we observed a high frequency of haplotypes containing the allele HLA-DRB1*04:11 in Lacandons, an allele that was found with a frequency of 0.3690, which is the highest frequency when compared to other neighboring indigenous groups^29,30^. Other authors^37^ have reported class II HLA alleles in the Lacandon (N = 162), and found an even higher frequency of HLA-DRB1*04:11 (Our study 0.3690 vs. ref. ^37^ 0.5740, *p* = 0.0002). Only two out of nine HLA class II blocks present in previous reports^37^ and in our study were found to be at different frequencies: HLA-DRB1*04:11~DQB1*03:02 (0.3688 vs. 0.5670, *p* = 0.0001) and HLA-DRB1*04:07~DQB1*03:02 (0.2398 vs. 0.0960, *p* = 0.0003). Several studies have suggested that HLA-DRB1*04:11 allele is of Native American MPA and is frequent in several Native North and South American populations including Tarahumaras from Mexico, Wayúu from Venezuela, Mataco-Wichi, Toba Pilaga and Eastern Toba from Argentina, and Cayapa from Ecuador^26,33,45,61,64,65^. Recent reports have associated the HLA-DRB1*04:11 allele with an increased susceptibility to pulmonary tuberculosis in Amazon Brazilian population^66^. One SNP position in LD with non-*DRB3*, non-*DRB5* haplotypes (i.e. the ones not linked with HLA-DRB1*04 allelic group, for instance) has been reported to be associated with a positive tuberculosis test in a recent study using genome-wide data paired with fine-mapping of the HLA region^67^. Although the significance is marginal, this finding further supports the involvement of the region in the susceptibility of tuberculosis. Also, the HLA-DRB1*04 allelic group alleles, highly prevalent in Lacandon Mayans, have been suggested to have a protective effect against hepatitis B virus infection^68^.

A high frequency of “rare alleles or haplotypes” may be present due to genetic isolation and small population number, both of which increase the effects of genetic drift. Class I (ABF: 0.4847), class II (ABF: 0.1659) and CEH (ABF: 0.5786) haplotypes previously unreported in Native American population account for an important part of haplotypic diversity in the Lacandon population, and they point to a distinctive Native American root that was overlooked until recent times^49^. For instance, haplotype HLA-A*31:01~B*40:02~C*03:04 (H.F: = 0.1310) was previously reported only in East Asian populations such as Japanese^69,70^, Chinese^71^ and Malaysia Peninsular Chinese (data collected by Sulaiman Salsabil, available in^61^), but it was also reported in mixed-ancestry populations such as Mexico City^72^ and “Hispanics” from USA^73^. Its frequency in Lacandon Maya is the highest frequency ever reported for this haplotype which would be indicative of a Native American MPA haplotype that can be traced back to its original East Asian ancestry thousands of years ago. HLA-B*35~C*07 associations, although uncommon, have been previously reported in Native North American populations such as Mixtec and Mixe^29^ but also in some Asian populations such as Rakhine from Myanmar (data collected by Thu ZinZin, available in^61^). Again, Lacandon Maya exhibit the highest frequency (H.F.: 0.1659) of this haplotype ever reported for any population. The reasons why these associations have never been previously found in Native American human groups include biased sampling procedures, but may also reflect genetic drift or pathogen selection having led to the extinction of specific haplotypes in other Native American groups, or the elevation of specific haplotypes in the Lacandon.

It is important to note that a European haplotype is present as part of the top ten most frequent haplotypes in our sample: A*02:01~B*18:01~C*07:01~DRB1*11:04~DQB1*03:01, with 2.40% (Table 6). That haplotype has been reported in frequencies ranging from 0.91% to 7.32% in populations from eastern and Mediterranean regions from Europe, such as Albania, Macedonia, Greece, Bosnia and Herzegovina, Romania and Italy^71,74–78^. The only African haplotype present at least twice in our sample was A*68:02~B*53:01~C*04:01~DRB1*13:03~DQB1*02:02 (HF = 0.44%; Table 6). This class I and class II haplotype can only be found in mixed ancestry populations such as Mexicans from Mexico City^72^ and African Americans^73^, but the class I block is present in populations from sub Saharan Africa such as the Bandiagara from Mali^79^, the Nandi and Luo from Kenya^79,80^ and the multicultural Worcester region in South Africa^81^. These two examples of non-Native American haplotypes give account of admixture events consistent with the ancestries brought into the Americas by conquerors during the conquest wars period and the colonial times^82,83^, even in what is considered to be one of the most isolated Native American human groups^52,53^.

HLA molecules have an important biological role as Killer cell Immunoglobulin-like Receptor (KIR) ligands. KIR2DL1, KIR2DL2/3 and KIR3DL1 bind HLA-C2, -C1 and -Bw4 ligands respectively, resulting in inhibition of natural killer (NK) cell-mediated cytolysis. C2, C1 and Bw4 are all found on HLA class I molecules: C1 and C2 are exclusive to *HLA-C*, while Bw4 can be found on some *HLA-B* and some *HLA-A* molecules. Several physiological functions of Natural Killer (NK) cells in human immunity and reproduction depend upon diverse interactions between KIRs and their HLA class I ligands^84–87^. In most populations, HLA-Bw4 alleles (i.e. those carrying asparagine, aspartic acid or serine in amino acid residue 77 and isoleucine or threonine in amino acid residue 80) are present in nearly 50% of the haplotypes, which means that around 75% of the individuals of any given population should express a ligand for the KIR3DL1 receptor^88,89^. Only 0.0503 of the *HLA-B* alleles present in Lacandon Maya are HLA-Bw4 alleles, and most of them (B*44:02, B*44:03, B*49:01, and B*13:02) are of European MPA. Nonetheless, Lacandon Maya have a relatively high frequency (A.F.: 0.0568) of the Bw4^+^ *HLA-A* allotype A*24:14, which is known to inhibit and to educate NK cells through interaction with KIR3DL1^89,90^. In our Lacandon sample, therefore, approximately 10.7% of haplotypes carry a Bw4^+^ ligand. HLA-C2 alleles are also inhibitory KIR ligands^91^; HLA-C2 frequencies in humans range from <10% in certain East Asian populations to ~60% in Oceania native human groups and some African populations. In our sample, the C2 ligand is present on 0.2294 of the HLA-C alleles (Table 1). This level of HLA-C2 is consistent with other Native American populations in which HLA-C2 alleles are present in AF < 0.3 such as Barí (0.075) and Yucpa (0.214) from Venezuela’s tropical humid forests^35,36^, both of which share the same type of ecosystem with Lacandon Maya.

As expected, when PIC values observed in Lacandons (Table 8) are compared against those found in a mixed ancestry population^72^, diversity is lower in the Lacandon sample than in Mexico City. Nevertheless, Lacandon PIC values are higher than other Native American groups for the *HLA-A* locus (Lacandons: 0.8444; Guarani from Paraguay: 0.7810; Toba from Argentina: 0.7715; Terena from Brasil: 0.7540; Mixtec: 0.7525, Zapotec: 0.7400, and Mixe: 0.6800 from Oaxaca, Mexico) and *HLA-B* (Lacandons: 0.8227; Gila River Indian Community of Arizona: 0.8167; Tarahumara from Mexico: 0.8093; Toba from Argentina: 0.7895; Mixtec: 0.7592, Zapotec: 0.7742, and Mixe: 0.3750 from Oaxaca, Mexico). Lacandon Maya *HLA-A* diversity as measured by PIC is the highest among Native American groups, and Lacandon *HLA-B* diversity is the third highest, just below Pilaga from Argentina and Sioux from USA. Conversely, *HLA-DRB1* shows relatively low diversity in Lacandon, below that found in Native South American groups (Lacandons: 0.7555; Toba: 0.8580, Pilaga: 0.8328, and Eastern Toba: 0.8326 from Argentina; Mapuche from Chile: 0.8333; Uro from Peru: 0.7879) and some Native North American groups (Zapotec from Oaxaca, Mexico: 0.8512; Sioux from USA: 0.8439).

In what could be called the “class I/class II diversity paradox”, the Lacandon population exhibits a relatively low diversity in *HLA-DRB1* (globally one of the most variable regions in the human genome) compared to *HLA-A* and *HLA-B*. It is possible that the lower diversity of MHC class II genes in Native American populations (and, by extension, in mixed populations with high proportions of Native American ancestry) might result from the frequency increase of some alleles that provide efficient immune protection against highly prevalent extracellular pathogens in specific populations^6,7,15,19,72^.

Previous studies have demonstrated a positive correlation between HLA class I allele diversity and pathogen richness and a negative correlation between HLA class II diversity and pathogen richness, raising the possibility that HLA class I and class II genes undergo different types of evolutionary trajectory in response to pathogen selection^5,6^. However, Sanchez Mazas *et al*.^6^ found that the significance of both their observed positive correlation between HLA class I allele diversity and pathogen richness and the negative correlation between HLA class II diversity and pathogen richness disappeared when Native American and Taiwanese populations were removed from the dataset. Native American populations have low HLA class II diversity in a high pathogen environment (this in turn seems to have helped to generate the previously observed negative correlation between class II diversity and pathogen richness). Is it possible that low HLA class II diversity can be a form of adaptation to a high pathogen environment? The class II HLA evolutionary mechanisms proposed by Sanchez-Mazas *et al*.^6^ apply particularly to *HLA-DQA1* and *HLA-DQB1*, whereas as noted previously the Lacandon also exhibit relatively low diversity in *HLA-DRB1*. Certain HLA alleles appear to be promiscuous and are capable of binding an exceptionally large set of epitope peptide segments^15^. Since the HLA class II alleles commonly found in Native Americans (except for HLA-DRB1*16:02), and specifically those reported here for Lacandon Mayans, do not fall within the category of “promiscuous alleles” established by Manczinger *et al*.^15^, we can in principle hypothesize that selection events happening in recent times may have driven this genetic structure. Other non-promiscuous alleles include those of the HLA-DRB1*04 allelic group (accounting altogether for 70.22% of the total *HLA-DRB1* diversity observed in Lacandons), with some of them being associated with specific pathogens as discussed above. It is noteworthy that at least one allele of this allelic group has been implicated in resistance to the development of enteric fever caused by *Salmonella enterica*^92^ and that there is molecular evidence of this pathogen causing at least one outbreak after the conquest in a region not far away from Chiapas (i.e. the state of Oaxaca)^93^. New alleles, with a very specific peptide binding repertoire, might be the most efficient way to achieve resistance to specific pathogens, as high promiscuity may not be able to cope with the rise of novel pathogens^15,94^.

Sanchez Mazas *et al*.^6^ found a positive relationship between *HLA-B* genetic diversity and either pathogen score or viral score, once distance from Africa was taken into account. In our analysis, our fitted coefficients were consistent with a positive relationship, but we could not reject the null hypothesis of no relationship (Supplementary Tables 3 and 4). Sanchez Mazas *et al*. did not find evidence for a relationship between *HLA-DRB1* genetic diversity and either pathogen score or viral score. Within our dataset, the strongest signal of any relationship (albeit one which was not robust to a Bonferroni correction) was for a negative relationship between *HLA-DRB1* genetic diversity and viral score once distance from Africa is considered, in non-Native American populations. We found no evidence for a relationship between either distance from Africa or pathogen/viral scores and HLA diversity when the Native American populations were considered alone. This may be due to the small size of the dataset. A better picture may be drawn in the coming years when ancient DNA analyses are taken into account for studying the immunogenetic diversity of these and other populations before and after specific events that would have changed the immune challenges faced by Native Americans, especially after contact with so-called Old World populations.

It has been shown using a multilocus model of host–pathogen co-evolution with allele-specific adaptive immunity that if selection from a multi-epitope, strain-structured pathogen is maintaining associations between host recognition loci, alleles at those loci should not only be in linkage disequilibrium, but also exhibit non-overlapping associations^24^. Penman *et al*.^24^ showed in particular that pathogen selection has the potential to maintain non-overlapping associations between HLA loci despite the presence of recombination between those loci. They demonstrated that such long-range non-overlapping associations may be observed between HLA loci such as *HLA-B* and *HLA-DRB1* in a dataset of HLA haplotypes from the Hutterite population of South Dakota. We examined non-overlapping associations within Lacandon HLA haplotypes in an effort to identify similar possible signatures of pathogen selection (Figs. 3, 4 and Supplementary Fig. 3). In our dataset, the highest levels of non-overlap between HLA loci are observed between the physically close *HLA-B* and *-C* pair and the similarly physically close *HLA-DRB1* and *HLA-DRB3/4/*5. Such associations could be a result of pathogen selection, but given the likely low level of recombination between these loci we cannot rule out other population genetic processes. For *HLA-DRB1* and *HLA-DRB3/4/5*, epistasis with the *HLA-DRA* locus with which the gene products of *HLA-DRB1* or *HLA-DRB3/4/5* must interact may contribute to particularly high levels of non-overlap. Recent work has also revealed that haplotypic associations between *HLA-B* and *HLA*-*C* in particular may be driven by the different mechanisms by which the proteins they encode are likely to be able to participate in NK cell education^95^.

There was no evidence for non-overlapping associations spanning class I and class II HLA loci in the Lacandon dataset, which may suggest that the most recent forms of pathogen selection in the Lacandon population have acted on the class I and/or class II loci separately. Furthermore, due to the particularly devastating infectious disease events the Lacandon, and other Native American populations have experienced – in which many people have died in large epidemics^93,96–99^– it is unlikely that the patterns seen in this group may be the result of the long-term pathogen selection simulated in the original Penman *et al*. model^24^. It may be that the Lacandon population maintained a set of haplotypes as a consequence of millennia of co-evolution with prevalent American pathogens, and the pattern seen today is the consequence of a short-term disruption of that state. It has been recently found^100^ that class II genes show a signature for selection when comparing Native Americans from the same region in the northwest coast of North America before and after the contact with Europeans and the European-borne pathogens during the conquest and colonial period^98,99,101^. Our results further add to the discussion on whether HLA class II region could have been under similar selective pressure in Native Americans.

In summary, the Lacandon Maya represent one of the most homozygous and least diverse populations regarding HLA class II genes among Native American populations. It is possible that a history of infections, genocides and inbreeding contributed to this limited diversity in the HLA system. The relationship between genetic diversity of the HLA system and both pathogen and viral diversity, as well as its correlation with the geographic distance from Africa, may become clearer if data from previous time periods are only considered. Future work using ancient DNA approaches to study populations before specific historic and prehistoric periods (e.g. the conquest of the Americas or great human migrations), but also identifying pathogens that caused outbreaks and epidemics, may shed light on the actual diversity of the HLA system in human populations before and after epidemic and pandemic events that may have shaped the immunogenetic diversity of our species.

## Subjects and Methods

### Subjects

A total of 218 first-degree non-related Lacandon individuals [146 ♀ (64.2%) and 82 ♂ (35.8%); average age = 31.7 ± 17.4 years] were studied for a final number of 436 chromosomes. All participants were inhabitants from small villages located in the Lacandon jungle in the southern State of Chiapas, Mexico including: Lacanjá, Bethel, E. Lacandón, Metzabok, Na Há, San Javier, and Tumbo, all of them belonging to the municipality of Ocosingo (Fig. 1). We confirmed the Lacandon ancestry (parents and grandparents born in the same region) of all included individuals by questionnaire. Collection of blood samples and demographic data was performed according to the requisites of the Helsinki Declaration (2008) and the General Health Law of Mexico and following the protocol approved jointly by the Ethics in Research Committee and the Research Committee from the National Institute for Medical Sciences and Nutrition “Salvador Zubirán” (INCMNSZ). All subjects provided written informed consent for these studies, and they authorized the storage of their DNA samples. Informed consent was obtained from all participants. When participants were under 18 years at the time of the sample collection, informed consent was obtained from a parent and/or legal guardian.

### High resolution HLA typing by next generation sequencing

Genomic DNA was obtained from peripheral blood mononuclear cells using the QIAamp DNA mini kit (Qiagen^®^, Valencia, CA, USA). All samples were typed for 11 HLA loci utilizing low- and high-resolution methods. Low resolution typing was performed by a Luminex-based detection and typing method including PCR amplification and reverse oligonucleotide hybridization (LABType^®^ SSO Typing Tests, One Lambda Inc., Canoga Park, CA, USA). High-resolution HLA class I and class II typing was performed by a recently developed high throughput sequencing technique involving long range PCR of each HLA locus, shearing of DNA and high throughput NGS typing as previously described^102^. In addition, these samples were typed with a commercial kit MIA FORA^TM^ NGS FLEX HLA Typing Kit (Immucor, Norcross, GA, USA) that utilizes a similar method which applies enzymatic fragmentation instead of Covaris sonication. HLA genotypes were assigned utilizing the ad hoc MIA FORA software HT9v1 (Immucor, Norcross, GA, USA), provided by the manufacturer with the reference sequence ver. 3.2.25 of the IMGT-HLA database^103^. The HLA typing system we used covers the class I genes in their full sequence. With exception of *HLA-DPB1*, the class II loci were covered in their full sequence. The sequence fragments (at least 300 bases long each) were put in phase. Given the number of SNPs, we could put in phase the sequences of almost all alleles. There were no ambiguities in the class I or class II loci with exception of *HLA-DPB1*. We had virtually no ambiguities in DPB1 because more than 65 percent of the subjects were homozygous in DPB1 resulting from the high frequency of DPB1*04:02 alleles (the frequency for DPB1*04:02:01:01 and DPB1*04:02:01:02 was ≥0.8). Among the few ambiguities observed, all of them resulted from genotypes that include alleles with identical sequences in exon-2 that differ in exon-3 and cannot be placed in phase because of lack of informative SNPs. Almost all ambiguous *HLA-DPB1* genotypes (<2%) included DPB1*04:02; we assigned the likely genotype as the one including this allele. In summary, there were no ambiguities for all loci with exception of *HLA-DPB1*; the rate of ambiguity at *HLA-DPB1* was low because of low allelic diversity at this locus in this population; the most likely genotype was assigned on the basis of allele distributions in unambiguous genotypes.

### HLA blocks and haplotypes assignment

HLA allele and haplotype frequencies were obtained by gene counting; seventy of the 458 haplotypes were obtained by family segregation analysis, since they were obtained by HLA typing of 69 individuals from 36 known families (related individuals were excluded to avoid overrepresentation of alleles and haplotypes). In 160 individuals without available samples from close family members, frequencies for alleles, two-point, three-point, four-point and five-point associations were determined using direct counting using the computer program Arlequin ver. 3.5^104^ and further corroborated with Hapl-o-Mat^105^ using an Expectation-Maximization (EM) algorithm. Arlequin was also used to calculate HWE, OH and EH at a locus-by-locus level with 1 × 10^6^ steps in the Markov chain; *p*-values ≤ 0.05 indicated statistical difference between OH and EH and thus a deviation from HWE and confirmed with an independent analysis done with PyPop^106^ ver. 0.7.0 using the Ewens-Watterson homozygosity (EWH) test of neutrality (tested by Slatkin’s implementation of the Monte-Carlo approximation of the Ewens-Watterson exact test). Listed *HLA-B*~*C*, *HLA-DRB1*~*DQB1*, *HLA-DRB1*~*DRB3/4/5*, *HLA-DQB1*~*DQA1*, *HLA-DPB1*~*DPA1*, Class I and Class II blocks, conserved extended haplotypes (*HLA-B*~*C*~*DRB1*~*DQB1*) and their extension to the *HLA-A* locus were estimated by maximum likelihood methods based on the standardized delta (Δ′) between alleles of two loci and between the two blocks and/or the extension to the *HLA-A* region, as previously described^72,107^. Estimation of delta (Δ) and standardized delta (Δ′) values to measure linkage disequilibrium (LD), nonrandom association of alleles at two or more loci, and their statistical significance, were calculated using previously described methods^23,107^. We used the statistic parameter *t* to validate all Δ′ data adjusted by sample size and number of times that each allele appeared in the sample^72,108^. Only *t* values ≥ 2.0 were considered significant. Most probable ancestry (MPA) for each haplotype was determined based on the relative frequencies of the haplotypes in well-defined continental populations. Based on previously published approaches^109^, haplotypes regarded as Native American MPA were defined as those found in significant frequencies (H.F. ≥ 1.0%) in Native American populations such as Argentina Gran Chaco Eastern Toba, Xavantes from Central Brazil^64^, South or Central America Native Americans^70^, Yucpa from Venezuela^35^, Aleut from Bering Island^70,110^, Penutian from British Columbia^111^, Tarahumaras from Northern Mexico^26^, Mixe, Mixtec and Zapotec from Oaxaca State, Southern Mexico^29,32^, North American Natives^107^ from USA, and Yup’ik^112^ from USA. European MPA haplotypes were defined as those present in significant frequencies (H.F. ≥ 1.0%) in European human groups such as Bosnia and Herzegovina [from the *Deutsche Knochenmarkspenderdatei* (DKMS), Germany], Greece (from the DKMS), Croatia (from the DKMS), Romania (from the DKMS), Spain (from the DKMS)^71^, Italy^78^, Gorski Kotar from Croatia^113^, Northern Ireland^114^ and Ireland^115^. Haplotypes of African MPA were defined as those found in significant frequencies (H.F. ≥ 1.0%) in African or African-descent human groups such as Azoreans from Terceira Islands (data collected by Jacome Bruges Armas, available in^61^), USA African American^73^, Bandiagara from Mali^79,116^, Kampala from Republic of Uganda^79,117^, Nandi from Kenya^79^ and Zambians^79,118^. Asian MPA haplotypes were found in H.F. above 1.0% in Asian human groups like Nganasan and Ket from Lower Yenisey River/Taimyr Peninsula region (Siberia)^119^, Chinese (from the DKMS)^71^, Taiwanese^120^, Japanese^69^, Ivatan from Bantanes, Republic of the Philippines^121^, and Koreans^122^. Mixed-ancestry populations included USA^70,73^ and Mexican^72^ well-defined mixed ancestry human groups. With these results, we calculated the aggregate block frequency (ABF)^17,123,124^ for each ancestry for class I, class II and CEHs.

### Analysis of HLA genetic diversity and non-overlapping associations between HLA loci

The genetic diversity of each HLA locus was assessed by two previously described forensic parameters: PIC and PD^125,126^; computed using the PowerStat ver.1.2 spreadsheet (Promega Corporation, Fitchburg, WI, USA) as described previously^72^. PIC measures the strength of a genetic marker for linkage studies by indicating the degree of polymorphism of a locus. PIC > 0.5 is considered as highly polymorphic^125^. PD is defined as the probability of finding two random individuals with different genotypes for that locus in the studied population, and values higher than 0.8 indicate high polymorphism in the studied population context^126^.

Penman *et al*. have argued that certain types of non-overlapping association between HLA loci may be a signature of pathogen selection^24^. The previously developed ${f}_{adj}^{\ast }$ metric^24,127^ was calculated for each pairwise combination of HLA loci in the dataset, using 436 Lacandon haplotypes for which we had data for every HLA locus. Penman *et al*. used ${f}_{adj}^{\ast }$ to compare patterns of non-overlap between HLA-HLA pairs and HLA-non HLA pairs of loci. In the current study we have only data on Lacandon HLA loci. In order to provide a point of comparison for the ${f}_{adj}^{\ast }$ score of each locus pair we generated 5000 random permutations of the order of the 436 alleles at one of the loci in each pair, and re-calculated ${f}_{adj}^{\ast }$ for each set of randomized data We thus generated distributions of possible ${f}_{adj}^{\ast }$ scores for each pair of loci in the dataset that would be obtained if the alleles at those loci were associated entirely at random. We then calculated the difference between the ${f}_{adj}^{\ast }$ value calculated from the Lacandon dataset for each pair of loci and the mean of the ${f}_{adj}^{\ast }$ scores calculated from the randomized data for that pair of loci, then divided that difference by the standard deviation of ${f}_{adj}^{\ast }$ calculated from the randomized data. The resulting scores allowed us to rank the HLA pairs in order of how extreme a level of non-overlap they displayed relative to entirely random associations between the same alleles. There are many reasons why associations between HLA loci should not be entirely random, so we do not claim that a departure from randomness necessarily means selection has occurred. However, ranking the pairs of HLA loci by how much they each depart from a state of random association allows for a more meaningful assessment of which pairs of HLA loci are most strongly non-overlapping, since it accounts for the differing allelic diversity at each locus.

### Analyzing genetic relationships between populations

A PCA plot and a population phylogenetic tree were constructed for 180 populations (including the Lacandon group studied in this work) with *HLA-A*, *HLA-B* and *HLA-DRB1* low-resolution data from a worldwide population dataset (Supplementary Table 1) using the IBM SPSS Statistics 19 software (IBM Corporation, Armonk, NY, USA) for the PCA and POPTREEW^128^ to analyze the distribution of 115 human groups with high resolution HLA typing, including our Lacandon sample. We used D_A_ distance^129^ and a NJ clustering method to construct a population phylogenetic tree with bootstrapping (1200 replications). References for each population group included in the PCA and phylogenetic analysis are listed given in Supplementary Table 1.

### Assessment of the correlation between pathogen richness and HLA diversity

We used PIC as an estimator for genetic diversity for three HLA loci (*HLA-A*, *HLA-B* and *HLA-DRB1*) and conducted an analysis of HLA genetic diversity and pathogen species richness similar to that of Sanchez-Mazas *et al*.^6^. First, we tested the correlation between genetic diversity for each gene and the geographic distance from Africa. We maintained the five key geographic points suggested by the authors and modeled the distance (in Km) from Addis Ababa (as a suggested point of departure) to every one of the locations where the 122 populations analyzed in the previous point (with available high-resolution data) live today. In our dataset the same populations were used for the three genes analyzed. Distance was calculated with the Measure distance function of Google Maps^130^. Information on pathogen and viral richness was extracted from the GIDEON database^57^. In order to relate the level of HLA polymorphism within a population and the pathogen environment of this population, we compiled the number of infectious diseases present in all countries for which we had information on HLA genetic diversity (N = 115). To assess the effect of genetic drift/recent bottlenecks/selection on small sized, isolated populations, we ran the statistical analyses after excluding Native American populations, and also ran tests on Native American populations separately. The final datasets include, thus, the worldwide populations’ dataset, the Native American populations’ dataset and the worldwide non-Native American populations’ dataset.

We used general linear models to determine how the distance from Africa and either pathogen or viral richness explained the level of genetic diversity (as estimated with PIC) at each of the three loci, for each of the datasets previously listed. We logit-transformed the PIC values before carrying out the analyses in order to improve normality. Coefficients for the relationships between distance, pathogen score or virus score; their 95% confidence intervals and associated *p* values, and the R^2^ value for each model are shown in Supplementary Tables 3 and 4. We performed the analyses with and without outliers. Outliers were defined as populations for which the logit transformed PIC value at any of the three loci was further than 2 standard deviations away from the mean logit(PIC) value for that locus.

## Supplementary information

Supplementary material.

## Data Availability

All data from our sample sets, both frequencies and anonymized individual genotypes, can be found at The Allele Frequency Net Database website (www.allelefrequencies.net)^131^ (AFDN Population ID: 3666). Sequence data can be found at the European Nucleotide Archive under accession numbers: KU319387, KU233196, KU319386, KU319291, KU319274, KU319273, KU319277, KU319263, KU319282, KX825916, KU319262, KU319256, KU319255, KU319285, KU319287, KU319303, KU319304, KU319325, KU319272, and KX825915. Further information can be requested to J.G. and/or M.F.V.
