# Supplementary material for: Diversity of HLA Class I and Class II blocks and conserved extended haplotypes in Lacandon Mayans
Source: Sci Rep. 2020 Feb 24;10:3248. doi: 10.1038/s41598-020-58897-5 (PMC7039995; doi:10.1038/s41598-020-58897-5)
Supplement: Supplementary file 1 — Supplementary material. [file 41598_2020_58897_MOESM1_ESM.pdf]

Diversity of HLA Class I and Class II blocks and conserved extended haplotypes in Lacandon Mayans.

Rodrigo Barquera*, Joaquin Zuniga*, José Flores-Rivera*, Teresa Corona*, Bridget S. Penman*, Diana Iraíz Hernández-Zaragoza*, Manuel Soler*, Letisia Jonapá-Gómez, Kalyan C. Mallempati, Petra Yescas, Adriana Ochoa-Morales, Konstantinos Barsakis, José Artemio Aguilar-Vázquez, Maricela García-Lechuga, Michael Mindrinos, María Yunis, Luis Jiménez-Alvarez, Lourdes Mena-Hernández, Esteban Ortega, Alfredo Cruz-Lagunas, Víctor Hugo Tovar-Méndez, Julio Granados‡, Marcelo Fernández-Viña‡, Edmond Yunis‡.

* These authors contributed equally to the present work.

‡ **Addresses for correspondence:** Julio Granados: julgrate@yahoo.com.mx; Marcelo Fernandez-Viña: marcelof@stanford.edu; Edmond Yunis: edmond_yunis@dfci.harvard.edu.

Supplementary information

Supplementary Table 1: Descriptions and references for the populations used in the population genetics analyses.3

Supplementary Figure 1: Principal Component Analysis for 163 populations (including the Lacandon group studied in this work).....17

Supplementary Figure 2: Population phylogenetic tree built with 114 human groups (including Lacandon Mayan from this work).....18

Supplementary Figure 3: Plots for all HLA class I and class II non-overlapping associations between HLA alleles. ..19

Supplementary Figure 4: PIC values of the three HLA genes analyzed vs. the geographic distance from Africa......20

Supplementary Table 2: *HLA-A~B~C* block frequencies.....22

Supplementary Table 3: The relationship between PIC, distance from Africa and pathogen richness at three different HLA loci.....24

Supplementary Table 4: The relationship between PIC, distance from Africa and viral richness at three different HLA loci.....25

Supplementary Material: References. 26

Supplementary Table 1: Descriptions and references for the populations used in the population genetics analyses.

Sample set name	Population description	Abbreviation	N =	References
Albania	Sample from the Blood Bank Unit of the University Hospital Center of Tirana, Albania	Al	160	[1]
Algeria	Sample from the Blood Bank Unit of Hospital Central de L'Armee, Algeria	Dz	106	[2]
Argentina Gran Chaco Eastern Toba	Toba from Eastern Gran Chaco Province, Argentina	ArE	135	[3]
Argentina Gran Chaco Mataco Wichi	Mataco Wichi from Gran Chaco Province, Argentina	ArM	49	[3]
Argentina Gran Chaco Western Toba Pilaga	Pilaga from Western Gran Chaco Province, Argentina	ArP	19	[3]
Argentina Rosario Toba	Toba from Rosario, Argentina	ArT	86	Data collected by Cintia Yanina Marcos [4]
Armenia	Sample from Armenian Bone Marrow Donors Registry	Am	100	[5]
Australia Cape York Peninsula Aborigine	Aborigine of Cape York's Peninsula, Australia	AuC	103	[6]
Australia Kimberley Aborigine	Aborigine from Kimberley Australia	AuK	41	[6]
Australia Yuendumu Aborigine	Aborigine from Yuendumu, Australia	AuY	191	[6]
Austria	Austria [from the Deutsche Knochenmarkspenderdatei (DKMS), Germany]	At	1698	[7]

Sample set name	Population description	Abbreviation	N =	References
Azores Central Islands	Azores from Central Islands	AzC	59	Data collected by Jacome Bruges Armas [4]
Azores Oriental Islands	Azores from Oriental Islands	AzO	43	Data collected by Jacome Bruges Armas [4]
Azores Terceira Islands	Azores from Terceira Islands	AzT	130	Data collected by Jacome Bruges Armas [4]
Bering	Aleut from Bering Island (Russia)	Ber	85	[8]
Bolivia Aymara	Aymara from Bolivia	BoA	102	[9]
Bolivia Quechua	Quechuas from La Paz/Titikaka Lake, Bolivia	BoQ	80	[10]
Bosnia	Bosnia and Herzegovina (from the DKMS, Germany)	Ba	1028	[7]
Brazil Ivaí Kaingang	Kaingang from Ivaí, Paraná, Brazil	BrI	127	[11]
Brazil Río das cobras Guaraní	Guaraní from Río das Cobras, Paraná, Brazil	BrG	98	[11]
Brazil Río das cobras Kaingang	Kaingang from Río das Cobras, Paraná, Brazil	BrR	113	[11]
Guarani-Kaiowá	Guarani-Kaiowá from Mato Grosso do Sul State, Brazil	BrK	144	[12]
Guarani-Ñandeva	Guarani-Ñandeva from Mato Grosso do Sul State, Brazil	BrN	53	[12]
Brazil Terena	Terena Tribe from Brazil	BrT	60	[13]

Sample set name	Population description	Abbreviation	N =	References
Bulgaria	Sample of Bulgarian individuals	Bg	55	[14]
Burkina Faso Fulani	Fulani from Ziniare, Burkina Faso	BfF	49	[15]
Burkina Faso Mossi	Mossi from Ziniare, Burkina Faso	BfM	53	[15]
Burkina Faso Rimaibe	Rimaibe from Ziniare, Burkina Faso	BfR	47	[15]
Cape Verde NW	Cape Verde, Northwest	CvN	62	[16]
Cape Verde SE	Cape Verde, Southeast	CvS	62	[16]
Cameroon	Cameroon	Cm	548 (*HLA* class I) + 126 (*HLA-DRB1*)	[17,18]
Central African Republic	Sample of Central Africa Pygmies	Cf	36 (*HLA* class I) + 93 (*HLA-DRB1*)	[19,20]
Chile Mapuche	Mapuche from Chile	Cl	104	[21]
China	China (from the DKMS, Germany)	Cn	1282	[7]
China Han	Han from the Canton region, China	CnH	264	[22]
China Han 1000G	Han from southern China	CnHG	100	[23]
China Beijing Shijiazhuang Tianjian Han	Tianjin, Beijing, and Shijiazhuang from northern China	CnB	618	[24]

Sample set name	Population description	Abbreviation	N =	References
China Guangxi Region Maonan	Maonan from Huangjiang, Guangxi Province, China	CnM	108	[25]
China Jiangsu Han	Donors (Han ethnic) recruited into the CMDP Jiangsu Branch	CnJ	3238	[26]
China Lisu	Lisu from Yunnan Province, China	CnL	111	[27]
China Yunnan Bulang	Croatia (from the DKMS, Germany)	CnY	116	[28]
Colombia Wayu	Wayu from Guajira, Colombia	Co	48	[29]
Croatia	Croatia (from the DKMS, Germany)	Hr	2057	[7]
Czech Republic	Bone Marrow Registry from Czech Republic	Cz	106	[30]
Czech Republic	Bone Marrow Registry from Czech Republic	Cz2	5099	Data collected by the Czech National Marrow Donors Registry [4]
NW Eng	English from North Western England	En	298	[31]
Finland 1000G	Finnish from Findland (1000 genomes Project)	FiG	100	[23]
France	France (from the DKMS, Germany)	Fr	1406	[7]
Georgia	Svans from the Georgia	Geo	80	[32]
Germany	Germany	De	13 386	[33]

Sample set name	Population description	Abbreviation	N =	References
Germany	Sample from the DKMS, Germany	De2	39 689	Data collected by J Mytilineos [4]
Guatemala Maya	Mayan from Guatemala	GtM	132	[34]
Guinea Bissau	Sample from Guiné-Bissau	Gw	65	[16]
Greece	Greece (from the DKMS, Germany)	Gr	1894	[7]
India Andhra Pradesh Golla	Sample from the Golla community in Andhra Pradesh, India	InA	111	[35]
India Mumbai Maratha	Maratha from Mumbai, India	InM	91	[36]
India North pop 2	Ethnic group of Punjab, Haryana, Utter Pradesh, and Delhi, India	InN	72	[37]
Indonesia Java Western	Western Javanese from Indonesia	IdW	236	[38]
Indonesia Javanese	Sample of Western Javanese from Indonesia	IdJ	201	[38]
Iran Baloch	Baloch from Iran	Ir	100	[39]
Iraq	Iraqis from the region of Kurdistan, Iraq	Iq	209	Data collected by Rawand Al-Qadi [4]
Ire N	Sample from Northern Ireland	IeN	1000	[40]
Ire S	Sample from Ireland	IeS	250	[41]

Sample set name	Population description	Abbreviation	N =	References
Italy	Italy (from the DKMS, Germany)	It	1159	[7]
Italy Tuscany 1000G	Italian from Tuscany (1000 genomes Project)	ItG	90	[23]
Japan	Sample from the Central Japan area	Jp	371	[42]
Japan Ainu	Ainu from Hokkaido, Japan	JpA	50	[43]
Japan Tokio 1000G	Japanese from Tokyo (1000 genomes Project)	JpG	181	[23]
Jordan Amman	Jordanians from Amman, Jordan	Jo	146	[44]
Kenya Luhya 1000G	Luhya from Webuye, Kenia (1000 genomes Project)	KeG	90	[23]
Libya Cyrenaica	Cyrenaica from Libya	Ly	118	[45]
Macedonia	Macedonians from Macedonia	Mk	172	[46]
Madeira	Madeirans from the Madeira-Porto Santo Archipelago	PtM	173	[47]
Malaysia	Malays from the Malaysian Marrow Donor Registry	My	1445	[48]
Malaysia Jelebu Temuan	Malays from Jelebu	MyJ	25	[49]
Malaysia Kedah Baling Kensiu	Malays from Kedah	MyK	25	[49]

Sample set name	Population description	Abbreviation	N =	References
Malaysia Perak Grik Jehai	Malays Grik from Perak	MyP	25	[49]
Malaysia Sarawak Bau Bidayuh	Malays From Sarawak	MyS	25	[49]
Doggon from Bandiagara, Mali	Doggon from Bandiagara, Mali	Ml	138	[50]
Mex Tarahumara	Tarahumaras from northern Mexico	MTa	44	[51]
Mex Teenek	Teenek from Huasteca region, Mexico	MTe	55	[52]
Mex Mayos	Mayos from northwestern Mexico	MMa	60	[53]
Mex Mazatecan	Mazatecan from Oaxaca State, southern Mexico	MMz	89	[54]
Mex Mixe	Mixe from Oaxaca State, southern Mexico	MMx	55	[55]
Mex Mixtec	Mixtec from Oaxaca State, southern Mexico	MMi	103	[55]
Mex Zapotec	Zapotec from Oaxaca State, southern Mexico	MZa	90	[55]
Mex Nahuas	Nahuas from central Mexico	MNa	85	[56]
Mex Seri	Seris from Sonora State, northwestern Mexico	MSe	34	[57]
Mongolia	Khoton from Tarialan, Mongolia	Mn	85	[58]

Sample set name	Population description	Abbreviation	N =	References
Metalsa from Morocco	Metalsa from Nador, Morocco	MaM	100	59
Morocco Atlantic Coast Chaouya	Chaouya Moroccans from the Atlantic Coast	MaC	98	60
Morocco	Moroccans from Morocco	Ma	96	61
Mozambique	Sample from blood donors from Maputo, Mozambique	Mz	202	62
Myanmar Kayin	Kayin from Myanmar	MmK	44	Data collected by T Zin Zin,, M Aye Aye, H Saji [4]
Myanmar Bamar	Bamar from Myanmar	MmB	46	Data collected by T Zin Zin,, M Aye Aye, H Saji [4]
Netherlands	Netherlands (from the DKMS, Germany)	Nl	1374	7
New Caledonia	Melanesians from New Caledonia and Vanuatu	Nc	65	63
New Caledonia 2	Melanesians from New Caledonia	Nc2	54	64
New Zealand Maori	Sample from Maori population of the Victoria University of The Wellington DNA Bank, New Zealand	NzM	46	65
New Zealand Polynesian	Sample from Maori population of the Victoria University of The Wellington DNA Bank, New Zealand	NzP	21	65
Nigeria	Nigerians from the South African Bone Marrow Registry	Ng	274	66
Nigeria Yoruba 1000G	Yoruba from Ibadan, Nigeria (1000 genomes Project)	NgG	89	23

Sample set name	Population description	Abbreviation	N =	References
Norway Sami	Sample of Sami people from Finnmark or Troms, Norway	NoS	200	[67]
Norway	Norwegian Bone Marrow Donor Registry	No	576	[67]
Pakistan Parsi	Parsi from Karachi, Pakistan	Pk	91	[68]
Palestina	Palestinian from the Gaza Strip, Palestina	Ps	165	[69]
Papua New Guinea East New Britain Rabaul	Melanesians from Rabaul islands, Papua-New Guinea	Pg	60	[63]
Papua New Guinea Highlander	Highlanders from Papua-New Guinea	PgH	92	[70]
Paraguay Guarani	Guarani Native Americans from Caaguazu, Canendiyu, and Guairà	Py	40	[71]
Peru Lama	Lamas from Lama City, Peru	PeL	83	[72]
Peru Titikaka Lake Uro	Uros from Titikaka Lake, Peru	PeU	105	[73]
Philippines Ivatan	Ivatan from Bantanes, Philippines	Ph	50	[74]
Filipino from Luzon Island, Philippines	Filipino from Luzon Island, Philippines	PhL	94	[75]
Poland	Poland (from the DKMS, Germany)	Pl	20 653	[7]
Portugal	Portugal (from the DKMS, Germany)	Pt	1176	[7]

Sample set name	Population description	Abbreviation	N =	References
Romania	Romania (from the DKMS, Germany)	Ro	1234	[7]
Russia Bashkir	Bashkir from Chelyabinsk Region, Russia	RuB	146	[76]
Russia Chuvash	Chuvashian from Chuvash Republic, Russia	RuC	82	[77]
Russia S Ural	Russian from the Chelyabinsk Region, Russian South Urals	RuU	207	[76]
Russia Tartar	Tartar from the Chelyabinsk Region, Russian South Urals	RuA	135	[77]
Russia Tuva	Tuvans from Tyva Republic, Siberia	RuT	190	[78]
Russia Tuva pop 2	Tuvans from Tyva Republic, Siberia	RuT2	169	[79]
Rwanda	Sample of women from Rwanda	Rw	280	[80]
Samoa	Samoas from Tokelau islands	Ws	51 (HLA-Class I) + 29 (HLA-DRB1)	[81,82]
Sao Tome Angolar	Angolares from São Tomé	StM	32	[83]
Sao Tome Forro	Forros from São Tomé	StF	66	[83]
Saudi Arabia Guraiat and Hail	Guraiat and Hail from Saudi Arabia	SaG	213	Data collected by K Hussein [4]
Senegal	Senegals from Mandeka and Dakar, Senegal	Sn	165 (*HLA-A*) + 112 (*HLA-B/-DRB1*)	[84,85]

Sample set name	Population description	Abbreviation	N =	References
Singapore Riau Malay	Singapore from Riau Malay	Sg	132	[86]
South Africa Black	South Africans of African Ancestry	ZaB	200	[87]
South Africa Caucasian	South Africans of European Ancestry	ZaC	102	[87]
Zulu from South Africa	Zulu from Natal Province, South Africa	ZaZ	199 (HLA-Class I) + 88 (HLA-DRB1)	[87]
South Korea	Sample set from South Korea	Kr	485	[88]
South Korea	Sample from CHA Medical Center Cord Blood Bank, South Korea	Kr2	4128	[89]
Spain	Spain (from the DKMS, Germany)	Es	1107	[7]
Spain Ibiza	Sample from Ibiza, Spain	EsI	88	[90]
Spain Maj Jews	Sample of Jews from Majorca, Spain	Esj	103	[90]
Spain Murcia	Sample from Murcia Region, Spain	EsM	173	[91]
Spain N Cabuer	Cabuernigos from Cantabria, Spain	EsC	95	[92]
Spain Pas Valley	Sample from Pas Valleys, Spain	EsP	88	[92]
Sudan Mixed	Sample of mixed ancestry population from Shaigiya, Sudan	Sd	200	Data collected by Elamin NE, Horn PA, Elbashir MI, Elkhidir IM, Elghazali G, Blasczyk R [4]

Sample set name	Population description	Abbreviation	N =	References
Sweden	Swedish from Sweden	Se	966	Data collected by Bengtsson Mats [4]
Sweden Northern Sami	Northern Swedish Sami	SNS	154	[93]
Sweden Southern Sami	Southern Swedish Sami	SSS	130	[93]
Taiwan Ami	Ami from Taiwan, China	TwA	98	[94]
Taiwan Minnan	Minnan from Taiwan, China	TwM	102	[94]
Taiwan Tzu Chi	Cord blood units from the Tzu Chi Taiwan Cord Blood Bank, China	TwC	710	[95]
Taiwan Atayal	Sample from Taiwan region Atayal, China	TwT	106	[94]
Taiwan Bunun	Sample from Taiwan region Bunun, China	TwB	101	[94]
Taiwan Hakka	Sample from Taiwan region Hakka, China	TwH	55	[94]
Taiwan Puyuma	Sample from Taiwan region Puyuma, China	TwP	50	[94]
Taiwan Saisiat	Saisiat from Wufen / Nanchuang, Taiwan, China	TwS	51	[94]
Thailand	Sample of blood donors, Thailand	Th	142	Data collected by Dasnayanee Chandanayingyong [4]
Thailand	Donors from the Thai Stem Cell Donor Registry, Thailand	Th2	16807	[96]

Sample set name	Population description	Abbreviation	N =	References
Tunisia	Southern Tunisians from Tunisia	Tn	82	[97]
Tunisia Gabes	South-eastern Tunisians from Gabes	TnG	95	[98]
Turkey	Turkey (from the DKMS, Germany)	Tr	4856	[7]
UAE	Sample from United Arab Emirates	Ae	298	[99]
Uganda	Kampala from Uganda	Ug	161 (*HLA-Class I*) + 103 (*HLA-DRB1*)	[100,101]
UK	United Kingdom (from the DKMS, Germany)	UK	1043	[7]
UK 1000G	British from England and Scotland (1000 genomes Project)	UKG	96	[23]
USA Gila River	Sample from the Gila River Indian Community of Arizona, United States of America	USG	492	[102]
US Navajo	Navajo from Cañoncito, New Mexico	USN	43	[102]
USA Sioux	Sioux from South Dakota, United States of America	USS	302	[103]
US Utah EU 1000G	Individuals of Northern and Western European ancestry from Utah (1000 genomes Project)	UtG	111	[23]
US Yupik	Yup'ik Esquimos from Yukon, Alaska, USA	USY	252	[104]

Sample set name	Population description	Abbreviation	N =	References
Vanuatu Aniwa	Melanesian from Aniwa Island	VuA	84	[64]
Vanuatu Futuna	Melanesian from Futuna Island	VuF	82	[64]
Vanuatu Paama	Melanesian from Paama Island	VuP	94	[64]
Vanuatu Santo	Melanesian from Santo Island	VuS	292	[64]
Vanuatu Tanna	Melanesian from Tanna Island	VuT	128	[64]
Venezuela Yucpa	Yucpa from the Perija Range, Venezuela	VeY	73	[105]
Vietnam	Kinh from Hanoi, Vietnam	Vn	170	[106]
Zambia	Zambia	Zm	44 (*HLA Class I*) + 292 (*HLA-DRB1*)	[100,107]
Zimbabwe Harare Shona	Zimbabwe Harare Shona	Zw	230	[108]
Rapanui	Rapa Nui from Easter Island	Rn	48	[109]

N refers to the number of individuals analyzed.

Supplementary Figure 1: Zoom-in to the Native American cluster of the Principal Component Analysis for 163 populations (including the Lacandon group studied in this work).

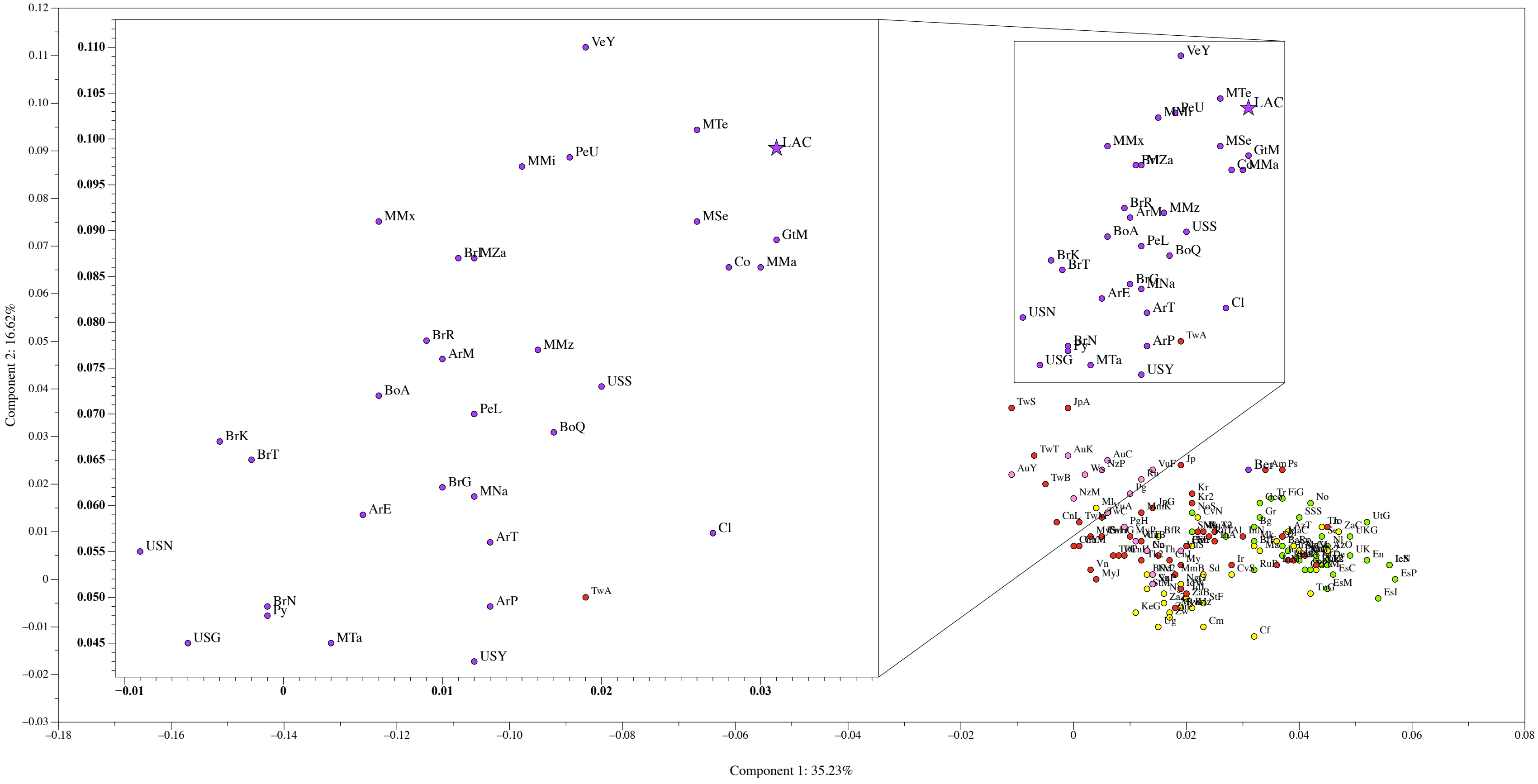

European populations are represented by green dots; African human groups correspond to yellow dots; red dots were assigned to Asian populations; Native American populations are represented by purple dots; Populations from Oceania are indicated with pink dots. Our Lacandon sample is represented by a purple star. The complete list of abbreviations is included in Supplementary Table 1.

Supplementary Figure 2: Population phylogenetic tree built with 114 human groups (including Lacandon Mayan from this work).

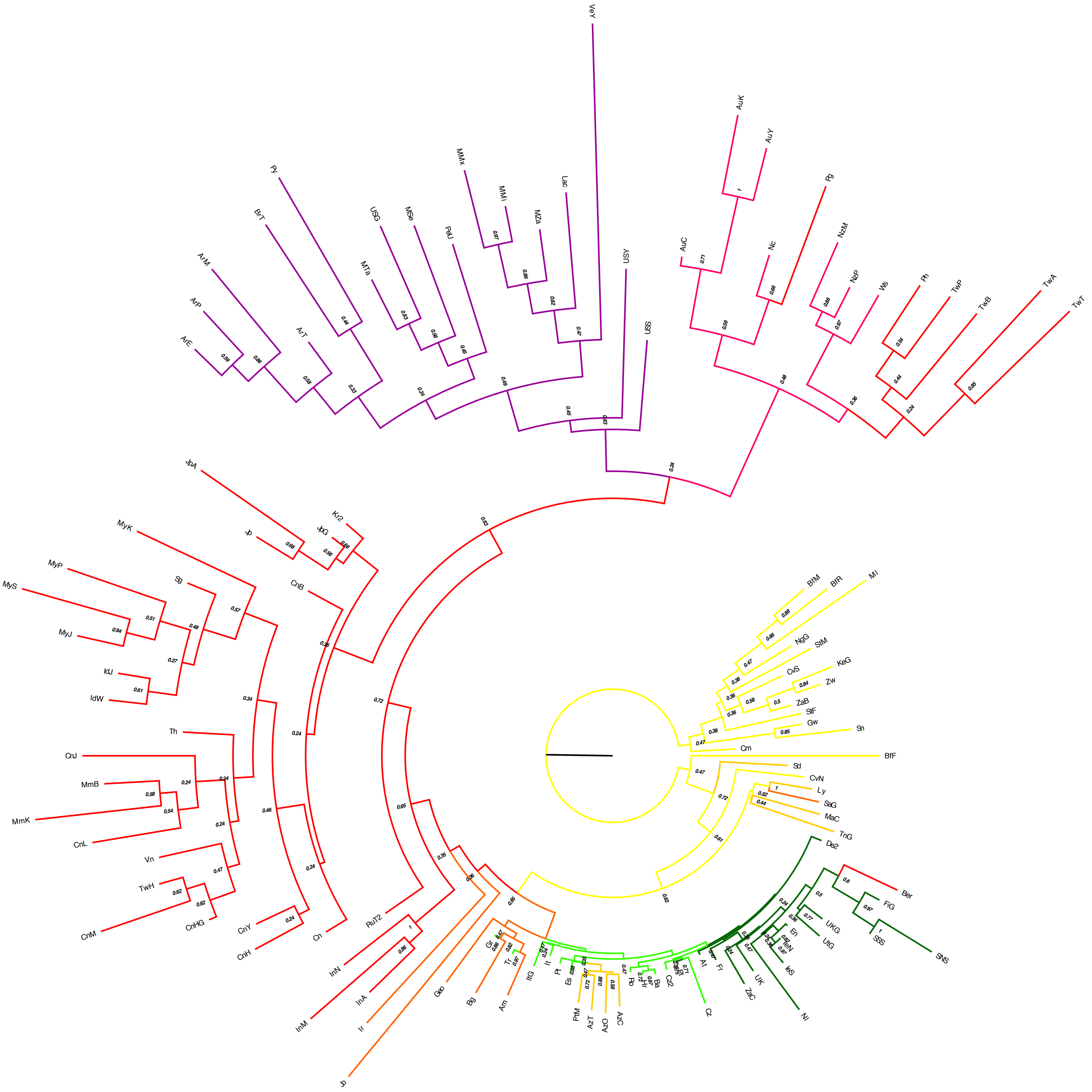

European populations are represented by green branches; African human groups correspond to yellow branches; red branches were assigned to Asian populations; Native American populations are represented by purple branches; populations from Oceania are indicated with pink branches. The complete list of abbreviations is included in Supplementary Table 1.

Supplementary Figure 3: Plots for all HLA class I and class II non-overlapping associations between HLA alleles.

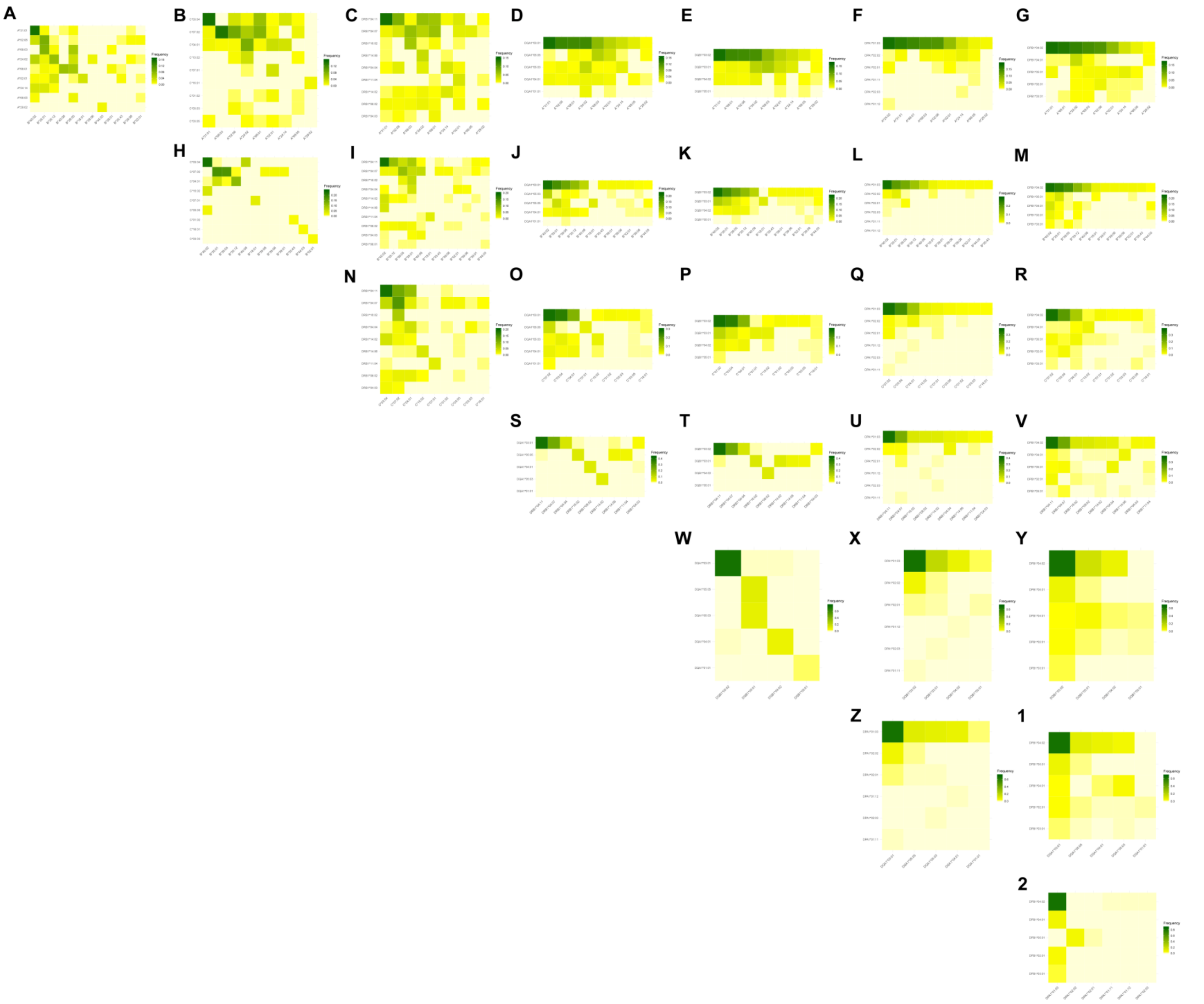

A. *HLA-A/-B.* B. *HLA-A/-C.* C. *HLA-A/-DRB1.* D. *HLA-A/-DQA1.* E. *HLA-A/-DQB1.* F. *HLA-A/-DPA1.* G. *HLA-A/-DPB1.* H. *HLA-B/-C.* I. *HLA-B/-DRB1.* J. *HLA-B/-DQA1.* K. *HLA-B/-DQB1.* L. *HLA-B/-DPA1.* M. *HLA-B/-DPB1.* N. *HLA-C/-DRB1.* O. *HLA-C/-DQA1.* P. *HLA-C/-DQB1.* Q. *HLA-C/-DPA1.* R. *HLA-C/-DPB1.* S. *HLA-DRB1/-DQA1.* T. *HLA-DRB1/-DQB1.* U. *HLA-DRB1/-DPA1.* V. *HLA-DRB1/-DPB1.* W. *HLA-DQA1/-DQB1.* X. *HLA-DQA1/-DPA1.* Y. *HLA-DQA1/-DPB1.* Z. *HLA-DQB1/-DPA1.* 1. *HLA-DQB1/-DPB1.* 2. *HLA-DPA1/-DPB1.*

Supplementary Figure 4: PIC values of the three HLA genes analyzed vs. the geographic distance from Africa.

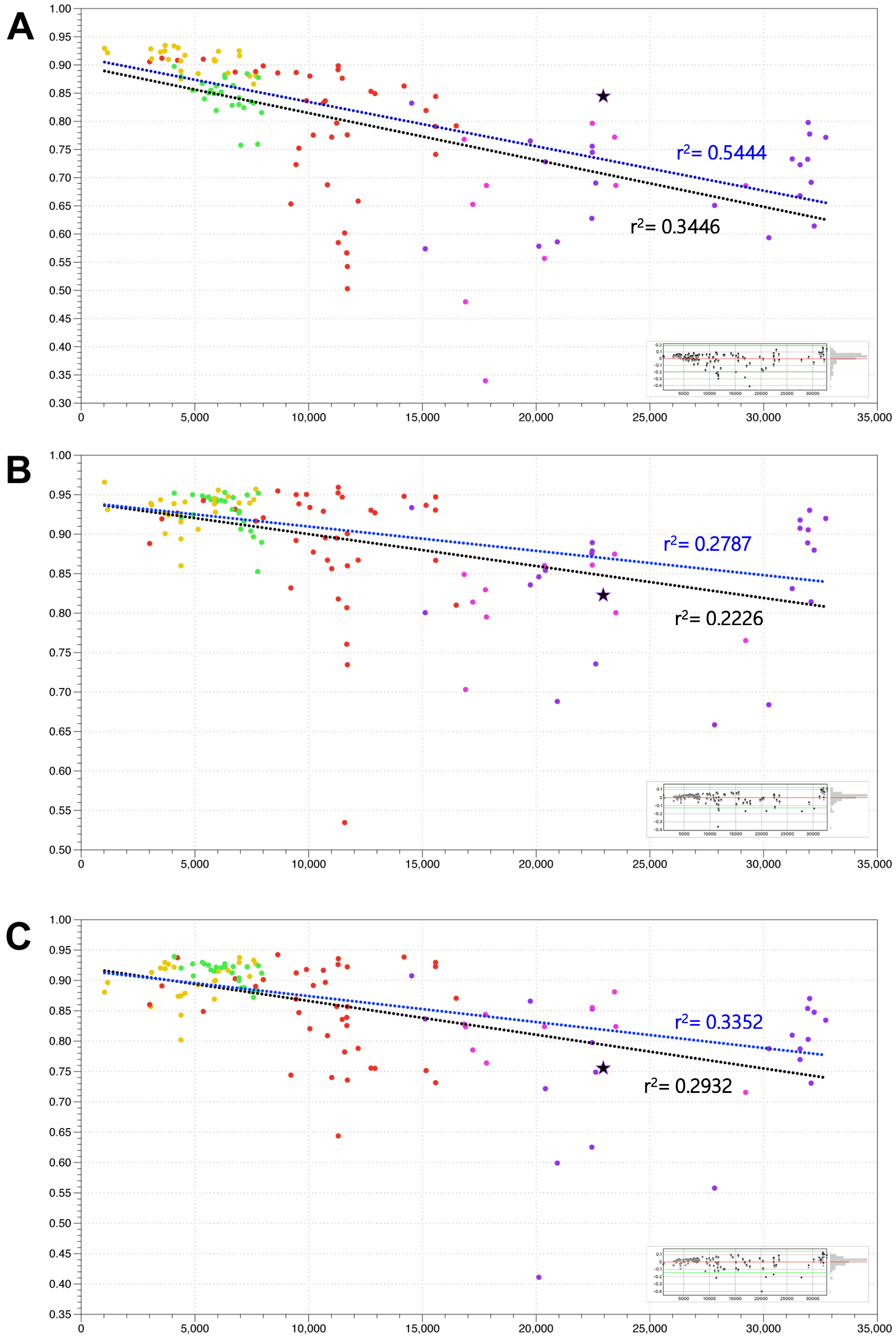

Distance from Africa (in Km) runs across the *x* axis and genetic diversity (as measured by polymorphism informative content, PIC) on the *y* axis. Black line depicts the linear regression taking all populations into account, while the blue represents the lineal regression without the outliers (σ=2). **A.** *HLA-A*. **B.** *HLA-B*. **C.** *HLA-DRB1*. Yellow dots represent African populations, green ones, European populations. Asian populations are depicted in red, Oceanian ones in pink and Native Americans in purple. Lacandon Mayans are represented by a star.

Supplementary Table 2: *HLA-A~B~C* block frequencies.

Block	MPA	H.F.	n (2N = 436)	Δ'	*p*	*t*
A*31:01~B*40:02~C*03:04	NatAm	0.1310	60	0.7500	< 0.0001	17.3
A*02:06~B*35:01~C*07:02	NatAm	0.0742	34	0.4491	< 0.0001	10.7
A*68:03~B*35:01~C*07:02	Unk	0.0742	34	0.4390	< 0.0001	10.6
A*24:02~B*35:12~C*04:01	NatAm	0.0611	28	0.5043	< 0.0001	9.5
A*68:01~B*40:08~C*03:04	Unk	0.0611	28	0.9596	< 0.0001	11.3
A*68:01~B*39:05~C*07:02	Unk	0.0546	25	0.2605	< 0.0001	7.2
A*68:03~B*39:05~C*07:02	Unk	0.0524	24	0.2560	< 0.0001	7.0
A*24:02~B*40:02~C*15:02	As	0.0306	14	0.7275	< 0.0001	7.1
A*02:01~B*18:01~C*07:01	Eu	0.0284	13	1.0000	< 0.0001	7.4
A*02:06~B*40:02~C*03:04	NatAm	0.0284	13	0.0079	0.8919	0.2
A*24:02~B*39:05~C*07:02	NatAm	0.0284	13	-0.0211	0.9260	-0.2
A*02:01~B*35:12~C*04:01	Unk	0.0262	12	0.1950	0.0001	5.0
A*24:14~B*40:02~C*03:04	Unk	0.0240	11	0.2788	0.0034	4.1
A*24:14~B*35:01~C*04:01	Unk	0.0218	10	0.4441	< 0.0001	6.2
A*24:02~B*39:06~C*07:02	Unk	0.0131	6	1.0000	< 0.0001	5.0
A*68:05~B*39:05~C*07:02	Unk	0.0131	6	0.4048	0.0011	3.9
A*24:02~B*35:01~C*04:01	Unk	0.0109	5	0.0658	0.5162	1.1
A*29:02~B*44:03~C*16:01	Eu	0.0109	5	1.0000	< 0.0001	4.5
A*24:02~B*40:02~C*03:04	NatAm	0.0087	4	-0.7590	0.0001	-6.8
A*68:01~B*39:01~C*07:02	Eu	0.0087	4	0.6094	0.0003	3.6
A*02:01~B*35:43~C*01:02	Unk	0.0066	3	0.5577	0.0001	3.2
A*02:06~B*39:08~C*07:02	Unk	0.0066	3	0.4186	0.0105	2.8
A*02:06~B*52:01~C*03:03	Unk	0.0066	3	0.5349	0.0029	3.1
A*68:03~B*35:12~C*04:01	Unk	0.0066	3	-0.5512	0.1040	-3.4
A*02:01~B*07:02~C*07:02	Eu	0.0044	2	0.6314	0.0007	2.7
A*02:01~B*40:02~C*03:05	Unk	0.0044	2	0.1708	0.1338	1.9
A*02:06~B*35:01~C*04:01	NatAm	0.0044	2	-0.3197	0.5449	-1.2
A*02:06~B*40:02~C*15:02	As	0.0044	2	-0.2063	0.7185	-0.7
A*24:02~B*35:17~C*04:01	Unk	0.0044	2	0.5913	0.0307	2.5
A*24:02~B*39:08~C*07:02	Unk	0.0044	2	0.1826	0.3438	1.4
A*24:02~B*40:02~C*03:05	NatAm	0.0044	2	0.0804	0.6295	0.8
A*24:14~B*35:12~C*04:01	Unk	0.0044	2	-0.2635	0.6364	-1.0
A*31:01~B*35:01~C*07:02	As	0.0044	2	-0.8378	0.0004	-6.9
A*31:01~B*35:08~C*04:01	Eu	0.0044	2	1.0000	0.0015	2.8
A*31:01~B*39:05~C*07:02	Unk	0.0044	2	-0.8333	0.0006	-6.8
A*31:01~B*40:02~C*03:05	Unk	0.0044	2	0.1000	0.5234	1.0
A*33:01~B*14:02~C*08:02	Eu	0.0044	2	1.0000	< 0.0001	2.8
A*68:01~B*35:01~C*04:01	Eu	0.0044	2	-0.3506	0.4951	-1.4
A*68:01~B*35:01~C*07:02	Unk	0.0044	2	-0.8157	0.0015	-6.5
A*68:02~B*53:01~C*04:01	Af	0.0044	2	1.0000	< 0.0001	2.8
A*68:03~B*39:01~C*07:02	Unk	0.0044	2	0.2228	0.1772	1.8
A*68:05~B*40:02~C*15:02	Unk	0.0044	2	0.1319	0.0232	2.3
Native American (NatAm) blocks ABF:					0.2162	
European (Eu) blocks ABF:					0.0808	
African (Af) blocks ABF:					0.0066	
Asian (As) blocks ABF:					0.1812	
Uncertain ancestry (Unk) blocks ABF:					0.5152	

H.F.: Haplotype frequency. MPA: most probable ancestry. Δ′: standardized linkage disequilibrium. Statistical significance value was set to $p < 0.00625$ after Bonferroni's correction. A value of $t > 2$ validates the LD for the block. *N* refers to the number of individuals analyzed.

Supplementary Table 3: The relationship between PIC, distance from Africa and pathogen richness at three different HLA loci.

As described in the Methods section, we used general linear models to explore the relationship between two predictor variables (distance from Africa and pathogen richness) and PIC at three HLA loci. We logit transformed the PIC values before fitting the model to improve normality. This table illustrates the results of fitting the model logit(PIC)=k.(distance from Africa) +m.(pathogen richness), for HLA-A, HLA-B and HLA-DRB1. We fitted each model using the complete dataset (All), and using only subsets of the data (Native American populations only, NAM and non-Native American populations, Non-NAM). The upper table shows results where all datapoints were included; the lower table shows results excluding outlier populations (defined as those where for at least one locus the logit(PIC) value was more than 2 standard deviations greater or lower than the mean logit(PIC) value for that locus). We took the Bonferroni corrected significance threshold (α) to be 0.005, since 9 hypotheses were tested on the global dataset (i.e. three HLA loci were each tested for relationships with distance from Africa; pathogen richness or viral richness). [$] indicates *p* values which are significant following this correction.

Complete dataset

		k (Gradient of relationship with distance from Africa)	Lower CI for k	Upper CI for k	P value for k	m (Gradient of relationship with pathogen richness)	Lower CI for m	Upper CI for m	P value for m	R^2 of model
HLA-A	All	-2.38E-05	-2.92E-05	-1.85E-05	9.70E-15[$]	0.000143	-0.00225	0.002539	0.905801	0.412855
	NAM	-8.35E-07	-1.42E-05	1.26E-05	9.03E-01	-3.12E-03	-0.00918	0.002948	0.321624	0.052882
	Non-NAM	-3.92E-05	-4.78E-05	-3.05E-05	2.39E-14[$]	7.19E-05	-0.00242	0.002566	0.954589	0.452778
HLA-B	All	-1.72E-05	-2.24E-05	-1.19E-05	1.79E-09[$]	0.000175	-0.00217	0.002518	0.882301	0.275163
	NAM	7.68E-06	-1.17E-05	2.71E-05	4.42E-01	-0.00043	-0.00921	0.008338	0.922913	0.03656
	Non-NAM	-2.71E-05	-3.53E-05	-1.89E-05	2.81E-09[$]	0.000332	-0.00203	0.002694	0.781578	0.306963
HLA-DRB1	All	-1.67E-05	-2.14E-05	-1.20E-05	1.11E-10[$]	-0.00275	-0.00485	-0.00065	0.010822	0.375246
	NAM	8.08E-07	-1.90E-05	2.06E-05	9.36E-01	-0.0062	-0.01516	0.002767	0.186986	0.099312
	Non-NAM	-2.11E-05	-2.83E-05	-1.38E-05	1.04E-07[$]	-0.00199	-0.00408	0.000101	0.062487	0.272909

Excluding outliers

		k (Gradient of relationship with distance from Africa)	Lower CI for k	Upper CI for k	P value for k	m (Gradient of relationship with pathogen richness)	Lower CI for m	Upper CI for m	P value for m	R^2 of model
HLA-A	All	-2.18E-05	-2.65E-05	-1.71E-05	4.46E-15[$]	0.000604	-0.00149	0.002701	0.569032	0.439755
	NAM	-3.24E-06	-1.60E-05	9.47E-06	0.620952	-0.00184	-0.00749	0.003814	0.529471	0.038506
	Non-NAM	-3.38E-05	-4.13E-05	-2.62E-05	7.29E-14[$]	0.000484	-0.00168	0.002648	0.658448	0.458534
HLA-B	All	-1.44E-05	-1.89E-05	-9.79E-06	9.03E-09[$]	0.000562	-0.00146	0.002583	0.582322	0.266597
	NAM	9.08E-06	-6.77E-06	2.49E-05	0.275315	-0.00065	-0.0077	0.006397	0.857608	0.095074
	Non-NAM	-2.34E-05	-3.05E-05	-1.63E-05	3.47E-09[$]	0.000694	-0.00133	0.002716	0.498236	0.319944
HLA-DRB1	All	-1.46E-05	-1.88E-05	-1.05E-05	2.38E-10[$]	-0.00145	-0.00329	0.000393	0.122074	0.352649
	NAM	-6.62E-06	-1.89E-05	5.65E-06	0.30327	-0.00317	-0.00863	0.002284	0.268459	0.127652
	Non-NAM	-1.97E-05	-2.68E-05	-1.26E-05	3.42E-07[$]	-0.0014	-0.00342	0.000616	0.171692	0.257768

Supplementary Table 4: The relationship between PIC, distance from Africa and viral richness at three different HLA loci.

As described in the Methods we used general linear models to explore the relationship between two predictor variables (distance from Africa and viral richness) and PIC at three HLA loci. We logit transformed the PIC values before fitting the model to improve normality. This table illustrates the results of fitting the model logit(PIC)=k.(distance from Africa) +m.(viral richness), for *HLA-A*, *HLA-B* and *HLA-DRB1*. We fitted each model using the complete dataset (All), and using only subsets of the data (Native American populations only, NAM and non-Native American populations, Non-NAM). The upper table shows results where all datapoints were included; the lower table shows results excluding outlier populations (defined as those where for at least one locus the logit(PIC) value was more than 2 standard deviations greater or lower than the mean logit(PIC) value for that locus). We took the Bonferroni corrected significance threshold (α) to be 0.005, since 9 hypotheses were tested on the global dataset (i.e. three HLA loci were each tested for relationships with distance from Africa; pathogen richness or viral richness). [$] indicates *p* values which are significant following this correction.

Complete dataset

		k (Gradient of relationship with distance from Africa)	Lower 95% CI for k	Upper 95% CI for k	P value for k	m (Gradient of relationship with pathogen richness)	Lower 95% CI for m	Upper 95% CI for m	P value for m	R^2 of model
HLA-A	All	-2.50E-05	-3.05E-05	-1.95E-05	3.91E-15[$]	0.005525	-0.00326	0.014311	0.215511	0.420339
	NAM	-8.01E-07	-1.35E-05	1.19E-05	0.901663	-0.01419	-0.03199	0.003617	0.131095	0.116969
	Non-NAM	-3.87E-05	-4.73E-05	-3.00E-05	4.72E-14[$]	0.005111	-0.00527	0.015497	0.332209	0.458066
HLA-B	All	-1.76E-05	-2.30E-05	-1.22E-05	2.34E-09[$]	0.002449	-0.00618	0.011081	0.575424	0.276946
	NAM	7.94E-06	-1.10E-05	2.69E-05	0.417524	-2.88E-05	-0.02671	0.026649	0.998319	0.036073
	Non-NAM	-2.69E-05	-3.52E-05	-1.87E-05	4.19E-09[$]	0.001811	-0.00807	0.011693	0.717541	0.307351
HLA-DRB1	All	-1.60E-05	-2.08E-05	-1.11E-05	1.67E-09[$]	-0.01017	-0.01792	-0.00242	0.010594	0.375446
	NAM	2.67E-06	-1.71E-05	2.25E-05	0.791807	-0.0143	-0.04212	0.013514	0.321383	0.061674
	Non-NAM	-2.19E-05	-2.92E-05	-1.47E-05	3.70E-08[$]	-0.00948	-0.01817	-0.00079	0.033308	0.28085

Excluding outliers

		k (Gradient of relationship with distance from Africa)	Lower 95% CI for k	Upper 95% CI for k	P value for k	m (Gradient of relationship with pathogen richness)	Lower 95% CI for m	Upper 95% CI for m	P value for m	R^2 of model
HLA-A	All	-2.29E-05	-2.77E-05	-1.81E-05	7.40E-16[$]	0.007197	-0.0006	0.014993	0.070053	0.454819
	NAM	-3.16E-06	-1.53E-05	8.94E-06	0.612595	-0.01046	-0.02797	0.007045	0.255944	0.100129
	Non-NAM	-3.30E-05	-4.06E-05	-2.54E-05	1.79E-13[$]	0.006732	-0.00224	0.015709	0.140559	0.470104
HLA-B	All	-1.45E-05	-1.92E-05	-9.82E-06	1.37E-08[$]	0.002296	-0.00532	0.00991	0.551339	0.266951
	NAM	9.14E-06	-6.42E-06	2.47E-05	0.263924	-0.0032	-0.02572	0.019323	0.782398	0.098022
	Non-NAM	-2.33E-05	-3.05E-05	-1.61E-05	5.64E-09[$]	0.001255	-0.00724	0.009751	0.770394	0.317163
HLA-DRB1	All	-1.43E-05	-1.86E-05	-1.01E-05	1.10E-09[$]	-0.00543	-0.01237	0.0015	0.123293	0.352557
	NAM	-5.23E-06	-1.79E-05	7.39E-06	0.425127	-0.00124	-0.01951	0.017033	0.895231	0.046129
	Non-NAM	-2.06E-05	-2.77E-05	-1.36E-05	9.32E-08[$]	-0.00899	-0.01735	-0.00064	0.035502	0.278188

Supplementary Material: References.

1. Sulcebe, G. *et al.* HLA allele and haplotype frequencies in the Albanian population and their relationship with the other European populations. *International Journal of Immunogenetics* **36**, 337–343 (2009).

2. Arnaiz-Villena, A. *et al.* HLA allele and haplotype frequencies in Algerians. Relatedness to Spaniards and Basques. *Human Immunology* **43**, 259–268 (1995).

3. Cerna, M. *et al.* Differences in HLA class II alleles of isolated South American Indian populations from Brazil and Argentina. *Hum Immunol* **37**, 213–220 (1993).

4. González-Galarza, F. F. *et al.* Allele frequency net 2015 update: New features for HLA epitopes, KIR and disease and HLA adverse drug reaction associations. *Nucleic Acids Research* **43**, D784–D788 (2015).

5. Matevosyan, L. *et al.* HLA-A, HLA-B, and HLA-DRB1 allele distribution in a large Armenian population sample. *Tissue Antigens* **78**, 21–30 (2011).

6. Gao, X., Mack, S. J., Currie, B., Asteal, S. & McCluskey, J. Anthropology/human genetic diversity population reports. Aboriginal Australian from Cape York, Australia. in *Immunobiology of the Human MHC: Proceedings of the 13th International Histocompatibility Workshop and Conference, Volume I* (ed. Hansen, J. A.) 624–62 (IHWG Press, 2007).

7. Pingel, J. *et al.* High-resolution HLA haplotype frequencies of stem cell donors in Germany with foreign parentage: How can they be used to improve unrelated donor searches? *Human Immunology* **74**, 330–340 (2013).

8. Moscoso, J. *et al.* HLA genes of Aleutian Islanders living between Alaska (USA) and Kamchatka (Russia) suggest a possible southern Siberia origin. *Molecular Immunology* **45**, 1018–1026 (2008).

9. Arnaiz-Villena, A. *et al.* Origin of Aymaras from Bolivia and their relationship with other Amerindians according to HLA genes. *Tissue Antigens* **65**, 379–390 (2005).

10. Martinez-Laso, J. *et al.* Origin of Bolivian Quechua Amerindians: Their relationship with other American Indians and Asians according to HLA genes. *European Journal of Medical Genetics* **49**, 169–185 (2006).

11. Luiza, M., Erler, P., Luz, R. & Sotomaior, V. S. The HLA polymorphsm of two distinctive South-American Indian tribes: The Kaingang and the Guarani. *Tissue Antigens* **41**, 227–237 (1993).

12. Petzl-Erler, M. L. & Tsuneto, L. T. Anthropology/human genetic diversity population reports. Guarani-Kaiowá Amerindians from Mato Grosso do Sul State, Brazil. in *Immunobiology of the Human MHC: Proceedings of the 13th International Histocompatibility Workshop and Conference, Volume I* (ed. Hansen, J. A.) 642–643 (IHWG

Press, 2007).

13. Lázaro, A. M. *et al.* Evolution of HLA-class I compared to HLA-class II polymorphism in Terena, a South-American Indian tribe. *Human Immunology* **60**, 1138–1149 (1999).

14. Ivanova, M. *et al.* HLA polymorphism in Bulgarians defined by high-resolution typing methods in comparison with other populations. *Tissue Antigens* **60**, 496–504 (2002).

15. Modiano, D. *et al.* HLA class I in three West African ethnic groups: Genetic distances from sub-Saharan and Caucasoid populations. *Tissue Antigens* **57**, 128–137 (2001).

16. Spínola, H., Bruges-Armas, J., Middleton, D. & Brehm, A. HLA polymorphisms in Cabo Verde and Guiné-Bissau inferred from sequence-based typing. *Human Immunology* **66**, 1082–1092 (2005).

17. Torimiro, J. N. *et al.* HLA class I diversity among rural rainforest inhabitants in Cameroon: Identification of A*2612-B*4407 haplotype. *Tissue Antigens* **67**, 30–37 (2006).

18. Pimtanothai, N., Hurley, C. K., Leke, R., Klitz, W. & Johnson, A. H. HLA-DR and -DQ polymorphism in Cameroon. *Tissue Antigens* **58**, 1–8 (2001).

19. Bruges Armas, J. *et al.* HLA class I variation in the West African Pygmies and their genetic relationship with other African populations. *Tissue Antigens* **62**, 233–242 (2003).

20. Renquin, J. *et al.* HLA class II polymorphism in Aka Pygmies and Bantu Congolese and a reassessment of HLA-DRB1 African diversity. *Hum Immunol* **58**, 211–222 (2001).

21. Rey, D. *et al.* HLA genetic profile of Mapuche (Araucanian) Amerindians from Chile. *Molecular Biology Reports* **40**, 4257–4267 (2013).

22. Trachtenberg, E. *et al.* HLA class I (A, B, C) and class II (DRB1, DQA1, DQB1, DPB1) alleles and haplotypes in the Han from southern China. *Tissue Antigens* **70**, 455–463 (2007).

23. Gourraud, P. A. *et al.* HLA diversity in the 1000 genomes dataset. *PLoS ONE* **9**, (2014).

24. Yang, G. *et al.* HLA-A, -B, and -DRB1 polymorphism defined by sequence-based typing of the Han population in Northern China. *Tissue Antigens* **67**, 146–152 (2006).

25. Ogata, S. *et al.* Polymorphisms of human leucocyte antigen genes in Maonan people in China. *Tissue Antigens* **69**, 154–160 (2007).

26. Qin Qin, P. *et al.* Distribution of human leucocyte antigen-A, -B and -DR alleles and haplotypes at high resolution in the population from Jiangsu province of China. *International Journal of Immunogenetics* **38**, 475–481 (2011).

27. Chen, S. *et al.* Origin of Tibeto-Burman speakers: Evidence from HLA allele distribution in Lisu and Nu inhabiting Yunnan of China. *Human Immunology* **68**, 550–559 (2007).

28. Shi, L. *et al.* Genetic link among Hani, Bulang and other Southeast Asian populations: Evidence from HLA -A, -B, -C, -DRB1 genes and haplotypes distribution. *International Journal of Immunogenetics* **37**, 467–475 (2010).

29. Silvera, C. *et al.* HLA genes in Wayu Amerindians from Colombia. *Immunological Investigations* **40**, 92–100 (2011).

30. Ivaskova, E. & Bendukidze, N. Anthropology/human genetic diversity population reports. Czech from Prague, Czech Republic. in *Immunobiology of the Human MHC: Proceedings of the 13th International Histocompatibility Workshop and Conference, Volume I* (ed. Hansen, J. A.) 597–598 (IHWG Press, 2007).

31. Alfirevic, A. *et al.* In silico analysis of HLA associations with drug-induced liver injury: Use of a HLA-genotyped DNA archive from healthy volunteers. *Genome Medicine* **4**, 51 (2012).

32. Sánchez-Velasco, P. & Leyva-Cobián, F. The HLA class I and class II allele frequencies studied at the DNA level in the Svanetian population (Upper Caucasus) and their relationships to Western European populations. *Tissue Antigens* **58**, 223–233 (2001).

33. Mullër, C. R., Ehninger, G. & Goldmann, S. F. Gene and Haplotype Frequencies for the on Over 13,000 German Blood Donors. *Human Immunology* **2003**, 137–151 (2003).

34. Gómez-Casado, E. *et al.* Origin of Mayans according to HLA genes and the uniqueness of Amerindians. *Tissue Antigens* **61**, 425–436 (2003).

35. Mack, S. J. *et al.* Anthropology/human genetic diversity population reports. Golla from Andrah Pradesh, India. in *Immunobiology of the Human MHC: Proceedings of the 13th International Histocompatibility Workshop and Conference. Volume I* (ed. Hansen, J. A.) 605–607 (IHWG Press, 2007).

36. Shankarkumar, U., Pawar, A., Ghosh, K., Bajpai, S. & Pazare, A. Human leucocyte antigen class II DRB1 and DQB1 associations in human immunodeficiency virus-infected patients of Mumbai, India. *International Journal of Immunogenetics* **37**, 199–204 (2010).

37. Rajalingam, R. *et al.* Distinctive KIR and HLA diversity in a panel of north Indian Hindus. *Immunogenetics* **53**, 1009–1019 (2002).

38. Yuliwulandari, R. *et al.* Association of HLA-A, -B, and -DRB1 with pulmonary tuberculosis in western Javanese Indonesia. *Human Immunology* **71**, 697–701 (2010).

39. Farjadian, S. *et al.* Molecular analysis of HLA allele frequencies and haplotypes in Baloch of Iran compared with related populations of Pakistan. *Tissue Antigens* **64**, 581–587 (2004).

40. Middleton, D., Williams, F., Hamill, M. A. & Meenagh, A. Frequency of HLA-B alleles in a caucasoid population determined by a two-stage PCR-SSOP typing strategy. *Human Immunology* **61**, 1285–1297 (2000).

41. Dunne, C., Crowley, J., Hagan, R., Rooney, G. & Lawlor, E. HLA-A, B, Cw, DRB1, DQB1 and DPB1 alleles and haplotypes in the genetically homogenous Irish population. *International Journal of Immunogenetics* **35**, 295–302 (2008).

42. Saito, S., Ota, S., Yamada, E., Inoko, H. & Ota, M. Allele frequencies and haplotypic associations defined by allelic DNA typing at HLA class I and class II loci in the Japanese population. *Tissue Antigens* **56**, 522–529 (2000).

43. Bannai, M. *et al.* Analysis of HLA genes and haplotypes in Ainu (from Hokkaido, northern Japan) supports the premise that they descent from Upper Paleolithic populations of East Asia. *Tissue Antigens* **55**, 128–139 (2000).

44. Sánchez-Velasco, P., Karadsheh, N. S., García-Martín, A., Ruíz De Alegría, C. & Leyva-Cobián, F. Molecular analysis of HLA allelic frequencies and haplotypes in Jordanians and comparison with other related populations. *Human Immunology* **62**, 901–909 (2001).

45. Galgani, A. *et al.* HLA-A, -B and -DRB1 allele frequencies in Cyrenaica population (Libya) and genetic relationships with other populations. *Human Immunology* **74**, 52–59 (2013).

46. Arnaiz-Villena, A. *et al.* HLA genes in Macedonians and the sub-Saharan origin of the Greeks. *Tissue Antigens* **57**, 118–127 (2001).

47. Arnaiz-Villena, A. *et al.* The peopling of Madeira archipelago (Portugal) according to HLA genes. *International Journal of Immunogenetics* **36**, 9–14 (2009).

48. Dhaliwal, J. S. *et al.* HLA-A, -B and -DR allele and haplo-type frequencies in Malays. *Asian Pacific Journal of Allergy and Immunology* **25**, 47–51 (2007).

49. Jinam, T. A., Saitou, N., Edo, J., Mahmood, A. & Phipps, M. E. Molecular analysis of HLA Class i and Class II genes in four indigenous Malaysian populations. *Tissue Antigens* **75**, 151–158 (2010).

50. Cao, K. *et al.* Anthropology/human genetic diversity population reports. Malian Admixed from Mali. in *Immunobiology of the Human MHC: Proceedings of the 13th International Histocompatibility Workshop and Conference, Volume I* (ed. Hansen, J. A.) 583–585 (IHWG Press, 2007).

51. García-Ortiz, J. E. *et al.* High-resolution molecular characterization of the HLA class I and class II in the Tarahumara Amerindian population. *Tissue Antigens* **68**, 135–146 (2006).

52. Vargas-Alarcon, G. *et al.* Distribution of HLA-B alleles in Mexican Amerindian populations. *Immunogenetics* **54**, 756–760 (2003).

53. Arnaiz-Villena, A. *et al.* HLA Genes in Mayos Population from Northeast Mexico. *Curr Genomics* **8**, 466–475 (2007).

54. Arnaiz-Villena, A. *et al.* HLA genes in Mexican Mazatecans, the peopling of the Americas and the uniqueness of Amerindians. *Tissue Antigens* **56**, 405–416 (2000).

55. Hollenbach, J. A. *et al.* HLA diversity, differentiation, and haplotype evolution in mesoamerican natives. *Human Immunology* **62**, 378–390 (2001).

56. Vargas-Alarcon, G. *et al.* Origin of Mexican Nahuas (Aztecs) according to HLA genes and their relationships with

worldwide populations. *Molecular Immunology* **44**, 747–755 (2007).

57. Infante, E., Aláez, C., Flores, H. & Gorodezky, C. Seri from Sonora, Mexico. Anthropology/human genetic diversity population reports. in *Immunobiology of the Human MHC: Proceedings of the 13th International Histocompatibility Workshop and Conference. Volume I* (ed. Hansen, J. A.) 633–634 (IHWG Press, 2007).

58. Munkhbat, B. *et al.* Molecular analysis of HLA polymorphism in Khoton-Mongolians. *Tissue Antigens* **50**, 124–134 (1997).

59. Piancatelli, D. *et al.* Anthropology/human genetic diversity population reports. Metalsa from Morocco. in *Immunobiology of the Human MHC: Proceedings of the 13th International Histocompatibility Workshop and Conference, Volume I* (ed. Hansen, J. A.) 594–595 (IHWG Press, 2007).

60. Canossi, A. *et al.* Correlation between genetic HLA class i and II polymorphisms and anthropological aspects in the Chaouya population from Morocco (Arabic speaking). *Tissue Antigens* **76**, 177–193 (2010).

61. Gómez-Casado, E. *et al.* HLA genes in Arabic-speaking Moroccans: Close relatedness to Berbers and Iberians. *Tissue Antigens* **55**, 239–249 (2000).

62. Assane, A. A. A. *et al.* Human leukocyte antigen-A, -B, and -DRB1 allele and haplotype frequencies in the Mozambican population: A blood donor-based population study. *Human Immunology* **71**, 1027–1032 (2010).

63. Gao, X., Bhatia, K., Trent, R. J. & Serjeantson, S. W. HLA-DR,DQ nucleotide sequence polymorphisms in five Melanesian populations. *Tissue Antigens* **40**, 31–37 (1992).

64. Maitland, K. *et al.* HLA class-I and class-II allele frequencies and two-locus haplotypes in Melanesians of Vanuatu and New Caledonia. *Tissue Antigens* **64**, 678–686 (2004).

65. Edinur, H. A. *et al.* HLA and MICA polymorphism in Polynesians and New Zealand Maori: Implications for ancestry and health. *Human Immunology* **74**, 1119–1129 (2013).

66. Steiner, D., Schlaphoff, T. & Borrill, V. HLA Study in Nigeria. *Tissue antigens* **68**, 2007 (2009).

67. Harbo, H. F. *et al.* Norwegian Sami differs significantly from other Norwegians according to their HLA profile. *Tissue Antigens* **75**, 207–217 (2010).

68. Mohyuddin, A. & Mehdi, S. Q. HLA analysis of the Parsi (Zoroastrian) population in Pakistan. *Tissue Antigens* **66**, 691–695 (2005).

69. Arnaiz-Villena, A. *et al.* The origin of Palestinians and their genetic relatedness with other Mediterranean populations. *Human Immunology* **62**, 889–900 (2001).

70. Mack, S. J. *et al.* Anthropology/human genetic diversity population reports. Highlander from Papua New Guinea. in *Immunobiology of the Human MHC: Proceedings of the 13th International Histocompatibility Workshop and Conference, Volume I* (ed. Hansen, J. A.) 621–623 (IHWG Press, 2007).

71. Benitez, O., Busson, M., Charron, D. & Loiseau, P. HLA polymorphism in a Guarani-Indian population from Paraguay and its usefulness for the Hispano-Indian admixture study in Paraguay. *International Journal of Immunogenetics* **38**, 7–11 (2011).

72. Moscoso, J. *et al.* HLA genes in Lamas Peruvian-Amazonian Amerindians. *Molecular Immunology* **43**, 1881–1889 (2006).

73. Arnaiz-Villena, A. *et al.* HLA genes in Uros from Titikaka Lake, Peru: Origin and relationship with other Amerindians and worldwide populations. *International Journal of Immunogenetics* **36**, 159–167 (2009).

74. Chu, C., Trejaut, J., Lee, H., Chang, S. & Lin, M. Ivatan from Bantanes, Philippines. Anthropology/human genetic diversity population reports. in *Immunobiology of the Human MHC: Proceedings of the 13th International Histocompatibility Workshop and Conference. Volume I* (ed. Hansen, J. A.) 611–615 (IHWG Press, 2007).

75. Erlich, H., Alejandrino, M., Pozzili, P., Panelo, A. & Bugawan, T. Population Anthropology/human genetic diversity population reports. Filipino from Luzon Island, Philippines. in *Immunobiology of the Human MHC: Proceedings of the 13th International Histocompatibility Workshop and Conference, Volume I* (ed. Hansen, J. A.) 620–621 (IHWG Press, 2007).

76. Suslova, T. A. *et al.* HLA gene and haplotype frequencies in Russians, Bashkirs and Tatars, living in the Chelyabinsk Region (Russian South Urals). *International Journal of Immunogenetics* **39**, 394–408 (2012).

77. Arnaiz-Villena, A. *et al.* HLA Genes in the Chuvashian Population from European Russia: Admixture of CentralEuropean and Mediterranean Populations. *Human Biology* **75**, 375–392 (2003).

78. Martinez-Laso, J. *et al.* HLA molecular markers in Tuvinians: A population with both Oriental and Caucasoid characteristics. *Annals of Human Genetics* **65**, 245–261 (2001).

79. Begovich, A. B. *et al.* Genetic variability and linkage disequilibrium within the HLA-DP region : analysis of 15 different populations. *Tissue Antigens* **57**, 424–439 (2001).

80. Tang, J. *et al.* Characteristics of HLA class I and class II polymorphisms in Rwandan women. *Experimental and clinical immunogenetics* **17**, 185–198 (2000).

81. Severson, L., Crews, D. & Lang, R. Samoans from American Samoan. Anthropology/human genetic diversity population reports. in *Immunobiology of the Human MHC: Proceedings of the 13th International Histocompatibility Workshop and Conference* (ed. Hansen, J. A.) 623–4 (IHWG Press, 2007).

82. Mack, S. *et al.* Evolution of Pacific / Asian populations inferred from HLA class II allele frequency distributions. *Tissue Antigens* **55**, 383–400 (2000).

83. Saldanha, N. *et al.* HLA polymorphisms in Forros and Angolares from São Tomé Island (West Africa): Evidence for the Population Origin. *Journal of Genetic Genealogy* **5**, 76–85 (2009).

84. Sanchez-Mazas, A., Steiner, Q. G., Grundschober, C. & Tiercy, J. M. The molecular determination of HLA-Cw

alleles, in the Mandenka (West Africa) reveals a close genetic relationship between Africans and Europeans. *Tissue Antigens* **56**, 303–312 (2000).

85. Andrien, M. & Dupont, E. HLA-B and -DRB1 allele frequencies in a population from Dakar, Senegal. *Human Immunology* **65**, 1071–1072 (2004).

86. Mack, S. J., Jani, A. J., Below, J. E., Saha, N. & Erlich, H. A. Anthropology/human genetic diversity population reports. Malay from Singapore. in *Immunobiology of the Human MHC: Proceedings of the 13th International Histocompatibility Workshop and Conference, Volume I* (ed. Hansen, J. A.) 615–616 (IHWG Press, 2007).

87. Paximadis, M. *et al.* Human leukocyte antigen class I (A, B, C) and II (DRB1) diversity in the black and Caucasian South African population. *Human Immunology* **73**, 80–92 (2012).

88. Lee, K. W., Oh, D. H., Lee, C. & Yang, S. Y. Allelic and haplotypic diversity of HLA-A, -B, -C, -DRB1, and -DQB1 genes in the Korean population. *Tissue Antigens* **65**, 437–447 (2005).

89. Huh, J. Y. *et al.* HLA-A, -B and -DRB1 polymorphism in koreans defined by sequence-based typing of 4128 cord blood units. *International Journal of Immunogenetics* **40**, 515–523 (2013).

90. Crespí, C. *et al.* HLA polymorphism in a Majorcan population of Jewish descent : Comparison with Majorca , Minorca , Ibiza (Balearic Islands) and other Jewish communities. *Tissue Antigens* **60**, 282–291 (2002).

91. Muro, M. *et al.* HLA polymorphism in the Murcia population (Spain): In the cradle of the archaeologic Iberians. in *Human Immunology* **62**, 910–921 (2001).

92. Sánchez-Velasco, P. *et al.* HLA alleles in isolated populations from North Spain : Origin of the Basques and the ancient Iberians. *Tissue Antigens* **61**, 384–392 (2003).

93. Johansson, Å., Ingman, M., Mack, S. J., Erlich, H. & Gyllensten, U. Genetic origin of the Swedish Sami inferred from HLA class I and class II allele frequencies. *European Journal of Human Genetics* **16**, 1341–1349 (2008).

94. Chu, C., Trejaut, J., Lee, H., Chang, S. & Lin, M. Population anthropology/human genetic diversity population reports. Ami from Hualien/Taitung; Minnan; Atayal from Wulai/Chenshih/Wufen; Bunun from Hsin-I/Taitung; Hakka from Hsinchu/Pintung; Puyuma from Peinan; Saisiat from Wufen / Nanchuang, Taiwan. in *Immunobiology of the Human MHC: Proceedings of the 13th International Histocompatibility Workshop and Conference, Volume I* (ed. Hansen, J. A.) 611–615 (IHWG Press, 2007).

95. Wen, S. H., Lai, M. J. & Yang, K. L. Human leukocyte antigen-A, -B, and -DRB1 haplotypes of cord blood units in the Tzu Chi Taiwan Cord Blood Bank. *Human Immunology* **69**, 430–436 (2008).

96. Kupatawintu, P. *et al.* HLA-A, -B, -DR haplotype frequencies in the Thai Stem Cell Donor Registry. *Tissue Antigens* **75**, 730–736 (2010).

97. Hajjej, A. *et al.* HLA genes in southern Tunisians (Ghannouch area) and their relationship with other Mediterraneans. *European Journal of Medical Genetics* **49**, 43–56 (2006).

98. Hajjej, A. *et al.* HLA class I and class II polymorphism in a population from south-eastern Tunisia (Gabes area). *International Journal of Immunogenetics* **38**, 191–199 (2011).

99. Santhosh, A. *et al.* A-B and A-B-DR haplotype frequencies in United Arab Emirates nationals. *Emirates Med J* **22**, 215–9 (2004).

100. Cao, K. *et al.* Differentiation between African populations is evidenced by the diversity of alleles and haplotypes of HLA class I loci. *Tissue Antigens* **63**, 293–325 (2004).

101. Okello, E. *et al.* Rheumatic heart disease in Uganda: The association between MHC class II HLA DR alleles and disease: A case control study. *BMC Cardiovascular Disorders* **14**, 2–6 (2014).

102. Williams, R. *et al.* Molecular variation at the HLA-A, B, C, DRB1, DQA1, and DQB1 loci in full heritage American Indians in Arizona: Private haplotypes and their evolution. *Tissue Antigens* **74**, 520–533 (2009).

103. Leffell, M. S. *et al.* HLA alleles and haplotypes among the lakota sioux: Report of the ASHI minority workshops, part III. *Human Immunology* **65**, 78–89 (2004).

104. Leffell, M. S. *et al.* HLA antigens, alleles and haplotypes among the Yup'ik Alaska natives: Report of the ASHI Minority Workshops, part II. *Human Immunology* **63**, 614–625 (2002).

105. Layrisse, Z. *et al.* Extended HLA haplotypes in a Carib Amerindian population: The Yucpa of the Perija Range. *Human Immunology* **62**, 992–1000 (2001).

106. Hoa, B. K. *et al.* HLA-A, -B, -C, -DRB1 and -DQB1 alleles and haplotypes in the Kinh population in Vietnam. *Tissue Antigens* **71**, 127–134 (2008).

107. Tang, J. *et al.* HLA-DRB1 and -DQB1 alleles and haplotypes in Zambian couples and their associations with heterosexual transmission of HIV type 1. *The Journal of infectious diseases* **189**, 1696–704 (2004).

108. Louie, L. *et al.* Population Anthropology/human genetic diversity population reports. Shona from Harare, Zimbabwe. in *Immunobiology of the Human MHC: Proceedings of the 13th International Histocompatibility Workshop and Conference, Volume I* (ed. Hansen, J. A.) 587–589 (IHWG Press, 2007).

109. Thorsby, E. The Polynesian gene pool: An early contribution by Amerindians to Easter Island. *Philosophical Transactions of the Royal Society B: Biological Sciences* **367**, 812–819 (2012).
